# The novel immunobiotic *Clostridium butyricum* S-45-5 displays broad-spectrum antiviral activity *in vitro* and *in vivo* by inducing immune modulation

**DOI:** 10.3389/fimmu.2023.1242183

**Published:** 2023-10-10

**Authors:** Kiramage Chathuranga, Yeseul Shin, Md Bashir Uddin, Jayoung Paek, W. A. Gayan Chathuranga, Yebin Seong, Lu Bai, Hongik Kim, Jeong Hwan Shin, Young-Hyo Chang, Jong-Soo Lee

**Affiliations:** ^1^ College of Veterinary Medicine, Chungnam National University, Daejeon, Republic of Korea; ^2^ Access to Genetic Resources and Benefit-Sharing (ABS) Research Support Center, Korea Research Institute of Bioscience and Biotechnology (KRIBB), Daejeon, Republic of Korea; ^3^ Department of Medicine, Sylhet Agricultural University, Sylhet, Bangladesh; ^4^ Department of Microbiology and Immunology, University of Texas Medical Branch, Galveston, TX, United States; ^5^ Research and Development Division, Vitabio, Inc., Daejeon, Republic of Korea; ^6^ Department of Laboratory Medicine, Inje University College of Medicine, Busan, Republic of Korea

**Keywords:** *Clostridium butyricum* S-45-5, probiotics, antiviral activity, interferon, influenza virus

## Abstract

*Clostridium butyricum* is known as a probiotic butyric acid bacterium that can improve the intestinal environment. In this study, we isolated a new strain of *C. butyricum* from infant feces and evaluated its physiological characteristics and antiviral efficacy by modulating the innate immune responses *in vitro* and *in vivo*. The isolated *C. butyricum* S-45-5 showed typical characteristics of *C. butyricum* including bile acid resistance, antibacterial ability, and growth promotion of various lactic acid bacteria. As an antiviral effect, *C. butyricum* S-45-5 markedly reduced the replication of influenza A virus (PR8), Newcastle Disease Virus (NDV), and Herpes Simplex Virus (HSV) in RAW264.7 cells *in vitro*. This suppression can be explained by the induction of antiviral state in cells by the induction of antiviral, IFN-related genes and secretion of IFNs and pro-inflammatory cytokines. *In vivo*, oral administration of *C. butyricum* S-45-5 exhibited prophylactic effects on BALB/c mice against fatal doses of highly pathogenic mouse-adapted influenza A subtypes (H1N1, H3N2, and H9N2). Before challenge with influenza virus, *C. butyricum* S-45-5-treated BALB/c mice showed increased levels of IFN-β, IFN-γ, IL-6, and IL-12 in serum, the small intestine, and bronchoalveolar lavage fluid (BALF), which correlated with observed prophylactic effects. Interestingly, after challenge with influenza virus, *C. butyricum* S-45-5-treated BALB/c mice showed reduced levels of pro-inflammatory cytokines and relatively higher levels of anti-inflammatory cytokines at day 7 post-infection. Taken together, these findings suggest that *C. butyricum* S-45-5 plays an antiviral role *in vitro* and *in vivo* by inducing an antiviral state and affects immune modulation to alleviate local and systemic inflammatory responses caused by influenza virus infection. Our study provides the beneficial effects of the new *C. butyricum* S-45-5 with antiviral effects as a probiotic.

## Introduction

Infectious viral diseases significantly impact the health of humans and livestock and the global economy (1). In the last two decades of the twenty-first century, there have been more than 10 major viral pandemics or epidemics in the human population caused by Coronaviruses, Myxoviruses, Filoviruses, Alphaviruses, and Flaviviruses. Simultaneously, zoonotic viruses consistently spread into humans from economically important livestock and other animals, demonstrating the importance of novel strategies to overcome future threats and maintain safe veterinary, medical, and public health practices (2). For instance, influenza, a major respiratory pathogen, has caused several epidemics or pandemics. Such as H1N1 (Spain in 1918), H3N2 (Hong Kong in 1968), H1N1pdm (Mexico in 2009) (3), and H7N9 (China in 2016). These viruses result in elevated rates of illness and death, particularly among vulnerable groups such as infants, the elderly, and individuals with compromised immune systems (4). Therapeutic agents and effective vaccines have been developed to prevent or treat viral diseases. Vaccination is the best way to prevent viral infections, but the gap between evolving epidemics and vaccine availability makes this approach less effective, particularly in the short term. Furthermore, there is no vaccine against emerging or re-emerging viruses. For example, the 2009 pandemic H1N1 virus had a high rate of mutation, and as a result, existing vaccines were unable to protect against emerging strains with different antigens (4). Therefore, it is necessary to develop alternative strategies to prevent virus infection, and alternative approaches have been proposed to reduce the incidence and severity of infectious viral diseases. During the last few decades, different research groups have demonstrated the immunomodulatory function of several gastrointestinal commensal microorganisms that have the ability to control the mucosal immune system (immunobiotics). These organisms have been proposed as a possible way to control influenza virus infection (4).

Probiotics can benefit host health by maintaining intestinal microecology, inhibiting the growth of pathogenic microorganisms, and enhancing local and systemic immune responses (5, 6). Probiotics such as *Lactobacillus reuteri* (7), *Lactobacillus rhamnosus* (8), *Bifidobacterium longum* (9), *Enterococcus faecalis* (10), *Lactiplantibacillus plantarum* (11), and *Basillus subtilis* (12) have been evaluated for their probiotic properties and have showed antiviral efficacy. Their antiviral actions include release of metabolites, direct interface with viruses, competition with cellular receptors to inhibit virus entry, enhancement of tight junctions between intestinal epithelial cells, and most importantly, modulation of mucosal and systemic immune responses (5). *In vivo*, oral administration of *L. rhamnosus* inhibits RSV infection by producing type I IFNs and by inducing interferon-stimulating genes such as Mx2, OAS1, IFNAR1, RNase L, OAS2, and IFITM3 (13). *L. gasseri* SBT2055 also showed potential to prevent respiratory syncytial virus infection by upregulating the expression of interferon-stimulating genes and IFNs (14). Moreover, oral administration of *B. longum* MM-2 induced the expression of cytokines (IL-12, IFN-γ, IL-18, and IL-2) in murine (9). Additionally, *in vivo* experiments involving *E. faecalis CECT7121* resulted in the robust activation of dendritic cells (DCs) and the subsequent secretion of elevated quantities of inflammatory cytokines (specifically IL-12, IL-6, and TNF-α) (10). Nevertheless, many probiotic strains remain to be discovered. Therefore, this study aims to address the existing gaps in understanding by focusing on finding novel probiotic bacteria that can potentially control virus infections by modulating immune regulation.

*Clostridium butyricum* is a strictly anaerobic, butyric acid-producing, spore-forming, Gram-positive bacteria in healthy animals and humans (15). Unlike *Lactobacillus and Bifidobacterium*, it can survive at lower pH levels, higher bile salt concentrations, and higher temperatures as an endospore-producing bacteria (16). *C. butyricum* has long been used as a probiotic in Asian countries such as Japan, Korea, and China (17). *C. butyricum* has been shown to promote *Lactobacillus* and *Bifidobacterium* growth and inhibit antibiotic-associated diarrhea in previous studies (16, 18). Oral administration of *C. butyricum* MIYAIRI 588 (CBM 588) strain enhanced the immune response in the respiratory tract of mice (19). The utilization of CBM 588 as a therapeutic approach effectively reduces intestinal inflammation by altering the host’s lipid metabolism through the upregulation of protectin D1, a lipid mediator renowned for its potent anti-inflammatory attributes (20). Moreover, CBM 588 prompts the development of IL-17-producing T cells, which significantly enhance mucins and tight junction proteins (TJs) within the colonic epithelium (21). Additionally, *C. butyricum* was reported to prevent acute experimental colitis in mice by inducing IL-10-producing macrophages in inflamed mucosa (22), and *Clostridium* cluster IV and XIVa are highly effective in decreasing inflammation via decreased production of IL-12 and INF-γ and increased production of anti-inflammatory cytokine IL-10 (23).

In this study, we isolated a new strain of *C. butyricum* from infant feces and evaluated its physiological characteristics and antiviral effects by the induction of an antiviral state and affecting immune modulation to alleviate local and systemic inflammatory responses caused by influenza virus infection *in vitro* and *in vivo*.

## Results

### Isolation and identification of *C. butyricum* S-45-5

Milk-white colonies that appeared after 48 h of incubation in RCM medium supplemented with 0.5% CaCO_3_ were selected to isolate and identify butyric acid bacteria strains from infant and adult feces. Initially, we screened 285 strains from infant feces and 223 strains from adult feces. The isolated strains were further identified by PCR amplification and sequencing of the 1,400-bp region of the 16S rRNA gene. As a result, we identified 109 strains from the 285 isolates from infant feces and 13 strains from the 223 isolates from adult samples as *Clostridium butyricum*. First, we screened 122 isolated *C. butyricum* strains to investigate their antiviral activity against virus-infected RAW264.7 cells. Among the selected *C. butyricum* strains, the *C. butyricum* S-45-5 strain isolated from infant feces was finally selected (data not shown) due to its prompt antiviral effect. Sequence result of this novel *C. butyricum* S-45-5 was deposited on the online depository, https://www.ncbi.nlm.nih.gov/, under the accession number GCA_003315755.1. In addition, the obtained sequence result is shown in **Supplementary Figure S1**. Next, the analysis commenced using the 1,424 base pair (bp) sequence from *C. butyricum* S-45-5 to conduct a comprehensive phylogenetic investigation. The results revealed that closed-related strains encompassed *C. butyricum* VPI3266 (98.9%), *C. butyricum* LQ25 (98.8%), and *C. butyricum* JCM 1391T (98.7%). Simultaneously, a maximum-likelihood tree revealed that the isolate clustered in a monophyletic clade within species *C. butyricum* (**Supplementary Figure S2A**). Upon diligent examination through the maximum-likelihood methodology, the phylogenetic evaluations established the isolate as an uncharted strain that belongs to the broader spectrum of *C. butyricum*. These notable conclusions were corroborated by analogous findings from neighbor-joining and minimum evolution analyses (**Supplementary Figure S2B**).

Additionally, the comprehensive genome sequencing endeavor yielded an impressive tally of 12,394,906 reads for the *C. butyricum* S-45-5 isolate, corresponding to a remarkably high coverage of 263 times. The counting arrangement exhibited a remarkable N50 value of 3,810,128 bp, complemented by an average counting size of 2,294,255 bp. This meticulous assemblage of the *C. butyricum* S-45-5 genome underwent detailed assessment via the CheckM tool, which confirmed a commendable genome completeness of 97.2%, with an inconspicuous 1.9% contamination rate. These facets collectively underscored the suitability of the genome for comprehensive scrutiny and analysis, as indicated by previous research (24). The aggregate size of the *C. butyricum* S-45-5 genome measured at 4,588,510 bp, while the DNA guanine–cytosine (G+C) content was quantified at 28.7 mol% (**Supplementary Table S1**). Employing automated multilocus sequence typing (autoMLST), the genome analysis garnered insight into the closest genomic analog, identifying *C. butyricum* DSM 10702T as the most akin (**Supplementary Figure S2C**). The ANI values between isolate *C. butyricum* S-45-5 and closely related species within the species *C. butyricum* ranged from 97.6% to 99.6%, and the dDDH values ranged from 78.8% to 96.3% (**Supplementary Table S2**). These findings imply that the identified strain represented a novel taxonomic entity within the broader category of *C. butyricum* species.

### Physiological characteristics of *C. butyricum* S-45-5

Acidic tolerance is an important criterion when selecting probiotic isolates to ensure their viability and functionality (25). Acid-resistance tests revealed that *C. butyricum* S-45-5 could exhibit survival rates of 60% at pH 2 and 90% at pH 3. The result demonstrates that *C. butyricum* S-45-5 could survive in the gastrointestinal tract’s acid condition (**Table 1**). Moreover, digestive enzyme tests showed that a new butyric-acid bacteria strain, *C. butyricum* S-45-5, inhibited amylase and proteases (**Table 2**). Bacterial resistance to bile salts in the intestine has generally been considered a prerequisite for colonization. The concentration of bile acids varies, ranging from approximately 8% in the gallbladder to approximately 0.2%–2% in the intestine. However, it is important to note that these values are not fixed, as levels of bile acids can vary between individuals due to factors like dietary intake (26). Since it has been found that bile salts can affect intestinal microflora and act as antimicrobial molecules (25), assessing their ability to tolerate bile salts before using *C. butyricum* as a probiotic is essential. The results of bile acid resistance tests indicated that *C. butyricum* S-45-5 was resistant enough to grow in a medium containing 3% Oxgall, indicating a solid resistance to bile acid (**Table 3**).

**Table 1:** Acid resistance test of *C. butyricum* S-45-5.

pH Value	Acid resistance
**pH1**	**−**
**pH2**	**+**
**pH3**	**+**
**pH4**	**++**
**pH5**	**++**
**pH6**	**++**

*Acid resistance percentage: (−, 0%) (w, <60%) (+, 60%–90%) (++, >90%).

**Table 2:** Digestive enzyme production assay of *C. butyricum* S-45-5.

Enzyme	Blank	S-45-5
**Amylase**	**−**	**+**
**Cellulase**	**−**	**+**
**Lipase**	**−**	**+**
**Protease**	**−**	**+**

Clear zone: + (Positive), − (Negative).

**Table 3:** Bile acid tolerance assay of *C. butyricum* S-45-5.

Oxgall (%)	Blank	S-45-5
**0.3**	**−**	**+**
**1**	**−**	**+**
**3**	**−**	**+**

+(Positive), −(Negative).

### Harmful bacteria antagonistic ability and antibiotics susceptibility tests

The potential probiotic strains should have possessed a good antibacterial activity against various gastrointestinal pathogenic bacteria. We evaluated the pathogenic bacteria antagonistic potential of *C. butyricum* S-45-5 *in vitro*. As shown in **Table 4**, *C. butyricum* S-45-5 culture supernatant (unadjusted pH = 4) showed antibacterial activity against *Pseudomonas aeroginosa*, *Enterobacter aerogenes*, and *Fusobacterium varium.* However, antibacterial activity against pathogenic bacteria was absent or much weaker in culture supernatants of the isolates adjusted to pH 6. We examined the antibiotic susceptibility of *C. butyricum* S-45-5 by analyzing the size of the inhibition zones in response to antibiotics. As shown in **Table 5**, *C. butyricum* S-45-5 generated inhibition rings > 20 mm diameter in response to penicillin (10 μg), tetracycline (30 μg), ampicillin (10 μg), and vancomycin (30 μg), indicating that *C. butyricum* S-45-5 is susceptible to these antibiotics. Inhibition rings < 20 mm in diameter were generated against kanamycin (30 µg), cephalothin (30 µg), clindamycin (2 µg), and gentamycin (10 µg), indicating lesser susceptibility. In addition, the *C. butyricum* S-45-5 was highly resistant to streptomycin (10 μg).

**Table 4:** Antimicrobial spectrum test of *C. butyricum* S-45-5.

Indicator Strain	S-45-5	
	pH4	pH6
***Enterobacter cloacea***	**−**	**−**
***Pseudomonas aeroginosa***	**+**	**−**
***Vibrio parahaemolyticus***	**−**	**−**
***Enterobacter aerogenes***	**+**	**−**
***Fusobacterium varium***	**+**	**−**

**Table 5:** Antibiotic susceptibility test of *C. butyricum* S-45-5.

Antibiotic	Clear zone (mm)
**Kanamycin**	**14**
**Penicillin**	**26**
**Cephalothin**	**19**
**Clindamycin**	**17**
**Tetracycline**	**35**
**Gentamycin**	**15**
**Streptomycin**	**8**
**Ampicillin**	**27**
**Vancomycin**	**28**

### The proliferation of lactic acid bacteria and analysis of organic acid metabolites

**Figure 1:**
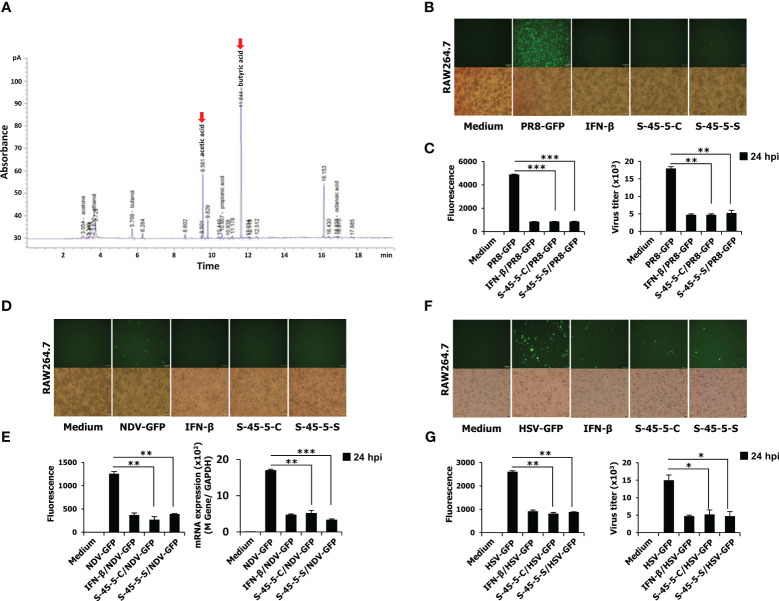
The organic acid production profile of *C. butyricum* S-45-5 and its antiviral activity in RAW264.7 cells. **(A)** HPLC analysis was carried out by using *C. butyricum* S-45-5 culture supernatant as the sample and ethanol, acetic acid, butanol, and butyric acid as standards. Employing helium as the carrier gas at a flowrate of 1 ml/min, the oven temperature was stepwise elevated from 50°C to 170°C at a rate of 10°C/min. The injector and detector temperatures were set to 250°C. RAW264.7 cells were added into 12-well cell culture plates, each well containing 2.5 × 10^5^ cells/well. Twelve hours later, cells were treated with PBS (medium, virus only), or 100 U/ml rmIFN-β, or *C. butyricum* S-45-5-Cell (1 × 10^6^ CFU/ml) or S-45-5-Sup (1 × 10^6^ CFU/ml). The medium was replaced with 1% fetal bovine serum (FBS) in Dulbecco’s modified Eagle’s medium (DMEM) 12 h later, and except for the medium, all other cells were infected with the green fluorescent protein (GFP) fused **(B, C)** H1N1 influenza virus (PR8-GFP, 1 MOI), **(D, E)** Newcastle disease virus (NDV-GFP, 2 MOI), or **(F, G)** Herpes simplex virus (HSV-GFP, 0.5 MOI). After 2 h, 10% FBS containing DMEM was added to the medium, and images were obtained following 24 h (200× magnification). GFP absorbance levels were measured by the GloMax multi-detection luminometer (Promega). A plaque assay was used to measure the virus titration in the supernatant and cells, and plaque-forming units were expressed (PFU). *Clostridium butyricum* S-45-5-Cell (S-45-5-C) and *Clostridium butyricum* S-45-5-Sup (S-45-5-S). GFP absorbance and virus titer are expressed as mean ± standard deviations (SD). Error bars indicate the range of values obtained from counting duplicates in three independent technical experiments (*p < 0.05, **p < 0.01, and ***p < 0.001 regarded as significant differences).

Lactic acid bacteria contribute to enormous health benefits to the host. Therefore, the ability of probiotic to grow lactic acid bacteria could be considered an advantage. The results of our study revealed that cultures of *C. butyricum* S-45-5 promoted growth of various strains of lactic acid bacteria. As shown in **Table 6**, the growth of the following bacteria was induced by indicated percentage: *L. plantarum* (15%), *L. salivarius* (135%), *L. rhamnosus* (25%), *L. sakei* subp. *sakei* (76%), and *B. catenulatum* (71%). Moreover, culture supernatants from *C. butyricum* S-45-5 promoted growth of *L. sakei* subp. *sakei*, *L. acidophilus*, and *B. catenulatum*. *Clostridium* species have been used preferably for butyric acid production, and *C. butyricum* has named after its capacity to produce butyric acid as the major end product during fermentation of sugars (27). Therefore, we analyzed the organic acid metabolites produced by *C. butyricum* S-45-5 using gas chromatography. As shown in **Figure 1A**, *C. butyricum* S-45-5 produces mainly butyric acid (56%), followed by acetic acid (35%). These results indicate that *C. butyricum* S-45-5 predominantly produce butyric acid as a representative organic acid metabolite.

**Table 6:** Lactic acid bacteria proliferation promotion effect of *C. butyricum* S-45-5.

Strain	S-45-5	
	Culture solution	Supernatant
***Lactobacillus plantarum***	**w**	**−**
***Lactobacillus reuteri***	**−**	**−**
***Lactobacillus salivarius***	**++**	**−**
***Lactobacillus rhamnosus***	**w**	**−**
***Lactobacillus paracasei* subsp. *paracasei***	**−**	**−**
***Lactobacillus sakei sub.sakei***	**+**	**w**
***Lactobacillus acidophilus***	**−**	**w**
***Lactobacillus casei***	**−**	**−**
***Enterococcus faecium***	**−**	**−**
***Bifidobacterium longum* subsp. *longum***	**−**	**−**
***Bifidobacterium catenulatum***	**+**	**w**
***Bifidobacterium longum subsp. infantis***	**−**	**−**
***Bifidobacterium bifidum***	**−**	**−**

*Bacteria proliferation promotion percentage: (−, 0%) (w, <50%) (+, 50%–99%) (++, >100%).

### *C. butyricum* S-45-5 inhibit viral replication in RAW264.7 cells

As mentioned, the *C. butyricum* S-45-5 strain was selected through antiviral activity screening against viruses. Before the antiviral assay, *in vitro* cytotoxicity assay was conducted. Indicated concentrations of *C. butyricum* S-45-5-Mix (*C. butyricum* S-45-5 live bacteria and culture supernatant) were treated into RAW264.7 or HEK293T cells, and cell viability at 24-h post-treatment was determined (**Supplementary Figures S3A–D**). The treatment of *C. butyricum* S-45-5 did not lead to notable cellular demise, except at the concentration of 5 × 10^7^ CFU/ml in RAW264.7 cells, which is 50 times higher concentration than that used for the *in vitro* antiviral experiments 1 × 10^6^ CFU/ml. Next, the *in vitro* antiviral effect of *C. butyricum* S-45-5 strain was investigated in detail by observing the amount of GFP expression in virus-infected cells as a measure of replication. RAW264.7 cells were treated with PBS (medium, virus only), or with 100 units/mL rmIFN-β, or with *C. butyricum* S-45-5 live bacteria (*C. butyricum* S-45-5-Cell) or with *C. butyricum* S-45-5 culture medium only (*C. butyricum* S-45-5-Sup). Compared with the untreated group, RAW264.7 cells treated with *C. butyricum* S-45-5-Cell and *C. butyricum* S-45-5-Sup exhibited a marked (>75%) reduction in GFP expression by influenza A virus (PR8-GFP) (**Figures 1B, C**), NDV-GFP (**Figures 1D, E**), and HSV-GFP (**Figures 1F, G**). Moreover, we confirmed *C. butyricum* S-45-5-mediated inhibition of virus replication by analyzing virus titers in the culture supernatants and infected cells. Interestingly, *C. butyricum* S-45-5-Cell and *C. butyricum* S-45-5-Sup treated RAW264.7 cells infected with PR8-GFP (**Figure 1C**, right graph), NDV-GFP (**Figure 1E**, right graph), and HSV-GFP (**Figure 1G**, right graph) produced significantly lower titers than control cells. The results indicate that the *C. butyricum* S-45-5-Cell and *C. butyricum* S-45-5-Sup inhibit replication of PR8-GFP, NDV-GFP, and HSV-GFP viruses in RAW264.7 cells.

### Effect of *C. butyricum* S-45-5 on cytokine production *in vitro*

**Figure 2:**
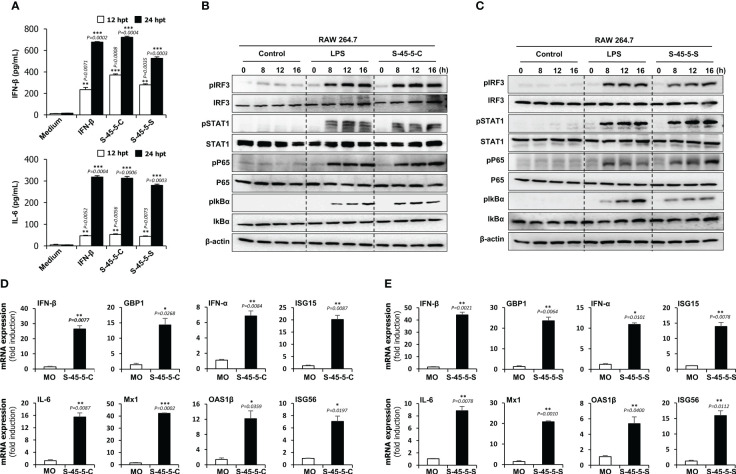
Induction of cytokine and phosphorylation of type-I IFN and NF-κB pathway-related signalling molecules by *C. butyricum* S-45-5 in RAW264.7 cell. **(A)** RAW264.7 cells were treated with PBS (medium), or 100 unit/ml rmIFN-β, or *C. butyricum* S-45-5-Cell (1 × 10^6^ CFU/ml) or S-45-5-Sup (1 × 10^6^ CFU/ml). Cell supernatants were harvested at 12 and 24 hour post-treatment and secreted IFN-β and IL-6 were measured by ELISA. **(B, C)** Activation of signal molecules present in type-I and NF-κB pathway was detected based on their phosphorylation status. RAW264.7 cells were treated with PBS, LPS (100 ng/ml) or *C. butyricum* S-45-5-Cell (1 × 10^6^ CFU/ml) or *C. butyricum* S-45-5-Sup (1×10^6^ CFU/ml) and cells were harvested at 0, 8, 12 and 16 hpt. Expression of the phosphorylated and non-phosphorylated forms of IRF3, STAT1, p65, and IkBα was analyzed time-dependently by immunoblotting. β-actin was used to confirm equal loading of proteins. **(D, E)** RAW264.7 cells were treated with *C. butyricum* S-45-5-Cell (1 × 10^6^ CFU/ml) or *C. butyricum* S-45-5-Sup (1 × 10^6^ CFU/ml). IFN-related antiviral gene mRNA level was measured at 24 hpt by RT-PCR and GAPDH was used for the normalization. Clostridium butyricum S-45-5-Cell (S-45-5-C), Clostridium butyricum S-45-5-Sup (S-45-5-S). All the values are expressed as mean + SD. Error bars indicate the range of values obtained from counting duplicates in three independent technical experiments. (* p < 0.05 ** p < 0.01 and *** p < 0.001 regarded as significant differences).

Cytokines play a pivotal role as essential mediators in the host’s immune response against pathogenic infections. They play an essential role in protection against viruses. To investigate the inhibitory effect of *C. butyricum* S-45-5 on the replication of RNA and DNA viruses, we measured the amounts of interferon-β (IFN-β) and IL-6 secreted by *C. butyricum* S-45-5-Cell- and *C. butyricum* S-45-5-Sup-treated RAW264.7 cells at 12- and 24-h post-treatment (hpt). Cells exposed to rmIFN-β (100 U/mL) were used as a positive control. As shown in **Figure 2A**, *C. butyricum* S-45-5-Cell- and *C. butyricum* S-45-5-Sup-treated cells secreted large amounts of IFN-β and IL-6 at 12 hpt and 24 hpt, similar to rmIFN-β-treated RAW264.7 cells. Hence, the live cells and culture supernatants derived from *C. butyricum* S-45-5 induce antiviral reactions within immune cells by promoting the expression of IFN and pro-inflammatory cytokines. This activation, in turn, drives innate antiviral responses, ultimately serving as a pivotal factor in restraining viral replication.

### *C. butyricum* S-45-5 mediated activation of the type I IFN and NF-κB signaling pathways

Both *C. butyricum* S-45-5-Cell and *C. butyricum* S-45-5-Sup induced secretion of IFN-β and IL-6 by immune cells. Therefore, we hypothesized that there might be a correlation between *C. butyricum* S-45-5 treatment and activation of IFN and NF-κB-inducing signaling pathways. To test this, we investigated the activation of the type I IFN and NF-κB signaling cascades in these cells upon exposure to *C. butyricum* S-45-5-Cell and *C. butyricum* S-45-5-Sup. To do this, we examined phosphorylation of IFN-related signaling molecules and NF-κB activation-related molecules in *C. butyricum* S-45-5-treated RAW264.7 cell lysates. As depicted in **Figures 2B, C**, both *C. butyricum* S-45-5-Cell and *C. butyricum* S-45-5-Sup demonstrated a notable enhancement in the phosphorylation levels of crucial signaling molecules within the type I IFN and NF-κB pathways, including IRF3, STAT1, p65, and IkBα (also see **Supplementary Figures S4A–D**, **Supplementary Figures S5A–H**). Specifically, the phosphorylation of IRF3, a pivotal marker in IFN signal transduction, and P65, a critical component of NF-κB transcription, exhibited initiation at 8 h post-treatment (hpt) and showed a progressive and substantial augmentation over the observation period. Furthermore, increased phosphorylation of STAT1 suggested transcriptional activation of ISGs, which control viral infection. Additionally, *C. butyricum* S-45-5-Cell- and *C. butyricum* S-45-5-Sup-treated RAW264.7 cells showed clear activation of NF-κB (p65, IkBα), leading to strong secretion of pro-inflammatory cytokines. These findings indicate that *C. butyricum* S-45-5 induces the secretion of pro-inflammatory cytokines and IFNs by activating NF-kB and type I IFN signaling pathways, thereby inhibiting the replication of RNA and DNA viruses in immune cells.

### *C. butyricum* S-45-5 induces expression of antiviral genes in RAW264.7 cells

So far, we demonstrated that *C. butyricum* S-45-5 trigger cytokine secretion and type I IFN signaling. Next, we asked whether these strains induce transcription of antiviral genes and ISG in RAW264.7 cells. A qPCR assay was performed to detect genes of interest (the primers are listed in **Supplementary Table S3**) in RAW264.7 cells treated with *C. butyricum* S-45-5. As shown in the **Figures 2D, E**, *C. butyricum* S-45-5-Cell- and *C. butyricum* S-45-5-Sup-treated RAW264.7 cells showed increased transcription of mRNA encoding IFN-β, IFN-α, IL-6, and ISGs GBP1, ISG15, Mx1, OAS1β, and ISG56 at 24 hpt. These data suggest that *C. butyricum* S-45-5-Cell and *C. butyricum* S-45-5-Sup upregulate transcription of important antiviral-related genes in RAW264.7 cells.

### Protection against diverse influenza A subtype infection by *C. butyricum* S-45-5 in BALB/c mice

**Figure 3:**
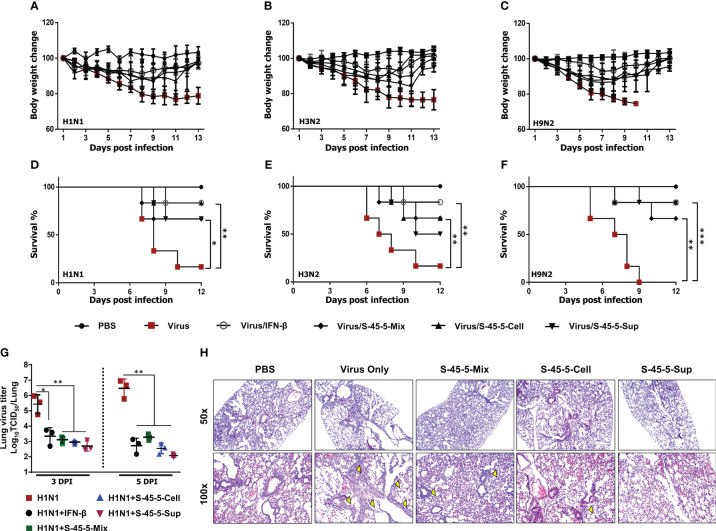
Oral administration of *C. butyricum* S-45-5 provides protection against lethal infection with divergent influenza A subtype in BALB/c mice. Six-week-old female BALB/c mice (n=6) were orally administrated with *C. butyricum* S-45-5-Mix (5 × 10^7^ CFU) or *C. butyricum* S-45-5-Cell (5 × 10^7^ CFU) or *C. butyricum* S-45-5-Sup (5 × 10^7^ CFU) in a total volume of 100 µl and control mice (PBS, Virus only, IFN-β) with 100 µl of PBS daily for 21 days as separate groups. Twelve hours before infection, mice in the positive control group were intranasally inoculated with 1,000 U of rmIFN-β. Except for the PBS group, all mice were intranasally infected with 2MLD_50_ of H1N1, or H3N2, or H9N2 influenza A subtypes. **(A–F)** Survival percentage and alterations in weight subsequent to the challenge were documented up to the 13th day post-infection (dpi). **(G)** Viral titers within the lung tissues of mice infected with H1N1 were quantified using TCID_50_ assays on the 3rd and 5th dpi. **(H)** HE (hematoxylin and eosin) staining for the lung sections was collected at 5 dpi. The arrows indicate inflammatory infiltration. Original magnification = 50× and 100×. (*p < 0.05, **p < 0.01, and ***p < 0.001) regarded as significant differences.

Previous studies report that oral administration of probiotic strains results in prolonged survival and reduced virus titers in the lungs of mice infected with influenza viruses (28). Therefore, to further define the prophylactic effects of *C. butyricum* S-45-5 strains, we orally administered *C. butyricum* S-45-5 live bacteria with culture medium (S-45-5-Mix, 5 × 10^7^ CFU), *C. butyricum* S-45-5-Cell (5 × 10^7^ CFU), or *C. butyricum* S-45-5-Sup (5 × 10^7^ CFU) to 6-week-old female BALB/c mice (n=6) (total volume 100 µL/mouse). Control mice received PBS (100 µL), virus alone (in 100 µL of PBS), or IFN-β alone (in 100 µL of PBS). Mice in control groups or *C. butyricum* S-45-5-treated groups were orally treated for 21 consecutive days, while mice in IFN-β group received their dose 12 h before virus infection. Next, mice were infected with a mouse-adapted lethal dose (2MLD_50_) of H1N1 (A/PR/8/34) or H3N2 (A/Philippines/2/08), or H9N2 (A/Chicken/Korea/116/2004), and morbidity and mortality were monitored daily up until 13 dpi. At approximately 5–6 days post-infection (dpi), most of the control group (virus only) mice exhibited severe clinical signs of respiratory disease (labored respiration and respiratory distress). Furthermore, the mice displayed reduced activity levels, a tendency to huddle, an arched posture, and disheveled fur. Additionally, there were substantial differences in body weight between the control and *C. butyricum* S-45-5-treated groups; control mice died by 9–10 dpi. Strikingly, most IFN-β (positive control)- and *C. butyricum* S-45-5-treated mice lost <20% of their body weight between 5 and 7 dpi, but began to recover their lost weight by 8 dpi, returning to their normal weight by 13 dpi (**Figures 3A–C**, **Supplement Figures S6A–C**). Moreover, groups that were orally inoculated with *C. butyricum* S-45-5 probiotic strains had significantly higher survival rates than the control groups (**Figures 3D-F**). The surviving mice in these groups showed no obvious clinical signs apart from mild weight loss. Influenza virus spreads efficiently via aerosols and replicates efficiently in the lungs (29). To determine the ability of *C. butyricum* S-45-5 to inhibit virus propagation in the lung, lung tissues were harvested from the H1N1-infected experimental group. Virus titration results revealed that H1N1 virus replicated efficiently in the lungs of the control group, with viral titers of 5.429 and 6.466 log TCID_50_/lung at 3 and 5 dpi, respectively. By contrast, the viral burden in the *C. butyricum* S-45-5-treated groups was several-fold lower than that of the control group. Specifically, the viral titer in the lungs of the *C. butyricum* S-45-5-Mix-treated group was 3.114 and 3.271 log TCID_50_/lung, that in the *C. butyricum* S-45-5-Cell-treated group was 2.956 and 2.548 log TCID_50_/lung, and that in the *C. butyricum* S-45-5-Sup-treated group was 2.699 and 2.075 log TCID_50_/lung at 3 dpi and 5 dpi, respectively. Titers in the rmIFN-β-treated group was 3.343 and 2.723 log TCID_50_/lung at 3 and 5 dpi, respectively (**Figure 3G**). The histopathological analysis of lung tissue demonstrated notable differences between H1N1-infected mice treated with PBS and non-infected mice receiving PBS alone, such as pronounced pathological alterations, including substantial alveolar wall thickening and infiltration of inflammatory cells into the alveoli. However, treatment with *C. butyricum* S-45-5 ameliorated these changes, resulting in reduced alveolar wall thickening and diminished inflammatory cell infiltration in the lung tissue (**Figure 3H**). These findings suggest that oral administration of *C. butyricum* S-45-5 inhibits viral replication in the lungs, thereby prolonging survival of mice infected with a lethal dose of influenza virus. Thus, *C. butyricum* S-45-5 provides broad protection from infection with diverse influenza A subtypes.

### Effect of *C. butyricum* S-45-5 on cytokine production in BALB/c mice

**Figure 4:**
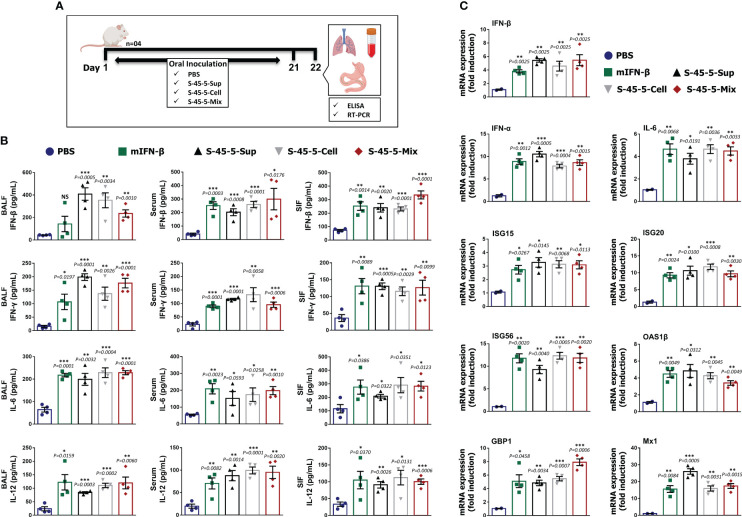
*C butyricum* S-45-5 induce interferons, pro-inflammatory cytokines, and antiviral-related gene expression in mice. **(A)** Schematic depiction of mouse experiment strategy. Six-week-old female BALB/c mice (n=4) were orally administrated with *C butyricum* S-45-5-Mix (5 × 10^7^ CFU), or *C butyricum* S-45-5-Cell (5 × 10^7^ CFU), or *C butyricum* S-45-5-Sup (5 × 10^7^ CFU) in a total volume of 100 µl and control mice with 100 µl of PBS daily for 21 days as separate groups. **(B)** On the next day (24 h following the last inoculation), BALF, serum, and small intestinal fluid (SIF) were collected and IFN-β, IFN-γ, IL-6, and IL12 were detected by ELISA using antibody-coated ELISA plate. **(C)** Lung tissues were collected simultaneously, and the transcription of ISGs was analyzed by real-time PCR. The ELISA tests were duplicated, and the data show representative means ± SD. Furthermore, error bars indicated in the mRNA expression represent the range of values obtained from groups of mice. (*p < 0.05, **p < 0.01, and ***p < 0.001 regarded as significant differences).

Our previous antiviral assays showed that *C. butyricum* S-45-5-treated immune cells produced large amounts of cytokines *in vitro*, and the *in vivo* results demonstrate that oral administration of mice with *C. butyricum* S-45-5 confers protective effects against influenza infection. Therefore, we next investigated the mechanism by which *C. butyricum* S-45-5 protects mice from the effects of different influenza viruses. To do this, we measured the amounts of IFN-β, IFN-γ, IL-6, and IL-12 in serum, BALF, and SIF at 24 h post-inoculation (**Figure 4A**). *C. butyricum* S-45-5-treated mice showed significantly higher production of IFN-β, IFN-γ, IL-6, and IL-12 in the BALF and blood compared with untreated mice, suggesting the induction of systemic innate immune response in BALB/c mice (**Figure 4B**). Moreover, mice inoculated with *C. butyricum* S-45-5 showed significantly higher levels of IFN-β, IFN-γ, IL-6, and IL-12 in the SIF (**Figure 4B**). These data suggest that *C. butyricum* S-45-5 mediates the antiviral response in BALB/c mice by eliciting expression of IFNs and pro-inflammatory cytokines, thereby triggering an antiviral state.

### Expression of antiviral genes in the lungs of mice orally administered *C. butyricum* S-45-5

To gain further insight into the possible mechanism by which *C. butyricum* S-45-5 strains induce antiviral responses, we performed transcriptional analyses of mRNA expression in lung homogenates after oral administration for 21 days (**Figure 4A**). Because microarray and RNA-sequencing results have demonstrated the existence of over 1,000 ISGs in diverse mammals (30), these ISGs contribute significantly to the host’s defense against viral infections. ISGs exert significant influence through key mechanisms such as impeding viral entry, obstructing virus replication and transcription, curtailing viral gene translation, and inhibiting viral protein synthesis (31). Here, we examined the transcription of six ISGs, IFNs, and IL-6 in lung homogenates of *C. butyricum* S-45-5-treated mice. Real-time PCR assay revealed a significant increase in all tested gene transcription in the lungs of *C. butyricum* S-45-5-treated mice over non-treated mice. Indeed, the expression of these genes were similar to that in the rmIFN-β-positive control group (**Figure 4C**). When comparing the acquired antiviral status by three different treatments, *C. butyricum* S-45-5-Sup-treated group showed higher or equal gene transcription over *C. butyricum* S-45-5-Cell or *C. butyricum* S-45-5-Mix-treated groups. It is important to note that ISG20, OAS1β, and Mx1 transcription was comparatively higher in *C. butyricum* S-45-5-Sup-treated group when compared with the *C. butyricum* S-45-5-Mix-treated group, which consists of both live cells and culture supernatant. A possible explanation for the observed result could be the efficient uptake of molecules from the supernatant rather than their interaction with intact bacteria, or the supernatant might stimulate a more immunogenic response than a synergistic effect of both live cells and supernatant. However, we recognize the need for further research to dissect the underlying mechanisms driving the observed result. Additional investigation, such as characterizing the composition of the supernatant and examining the specific host cellular responses, could provide valuable insights into this phenomenon. However, this acquired antiviral state upon *C. butyricum* S-45-5 treatment could be helpful to overcome the burden during influenza virus infection.

### *C. butyricum* S-45-5 affects immune modulation to alleviate local and systemic inflammatory responses caused by influenza virus infection in BALB/c mice

**Figure 5:**
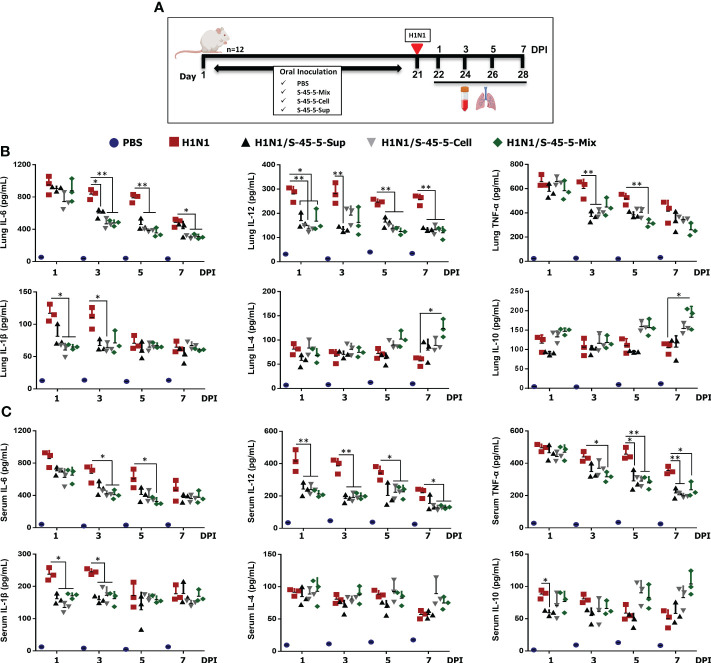
Effect of *C butyricum* S-45-5 on serum and lung pro-inflammatory cytokine and anti-inflammatory cytokine secretion at 1, 3, 5, and 7 day post-infection (DPI). **(A)** Schematic depiction of mouse experiment strategy. Six-week-old female BALB/c mice (n=12) were orally administrated with *C butyricum* S-45-5-Mix (5 × 10^7^ CFU), or *C butyricum* S-45-5-Cell (5 × 10^7^ CFU), or *C butyricum* S-45-5-Sup (5 × 10^7^ CFU) in a total volume of 100 µl and control mice (PBS, H1N1) with 100 µl of PBS daily for 21 days as separate groups. Except for the PBS group, all mice were intranasally infected with 1MLD_50_ of the H1N1 influenza A subtype. Lung tissue and serum were collected at 1, 3, 5, and 7 dpi (n=3). Secreted IL-12, IL-6, IL-1β, TNF-α, IL-4, and IL-10 in **(B)** serum and **(C)** lung homogenate were measured by ELISA. Data expressed as mean ± standard deviations (SD). Error bars indicate the range of values obtained from counting duplicates in three independent samples (*p < 0.05, **p < 0.01, and ***p < 0.001 regarded as significant differences).

Infection with pathogenic influenza virus leads to excessive production of pro-inflammatory cytokines, which in turn leads to aggressive inflammatory responses. If anti-inflammatory responses by the host do not counter these responses, a cytokine storm can occur. Cytokine storm is one of the main causes of mortality during influenza-virus infection (32). To better understand the effect of probiotic strain *C. butyricum* S-45-5 on immune modulation upon influenza infection, we measured local (lung) and systemic (serum) expression of pro-inflammatory (IL-6, IL-12, TNF-α, and IL-1β) and anti-inflammatory (IL-4, IL-10) cytokines at four different days following influenza infection (**Figure 5A**). As shown in the **Figures 5B, C**, mice infected with the influenza virus showed significantly higher secretion of cytokines in lung homogenates and serum than non-infected mice. Interestingly, administration of *C. butyricum* S-45-5-Sup led to a significant reduction in the amounts of IL-6, IL-12, and TNF-α in lung homogenates at 3 dpi, at 1, 3, 5, and 7 dpi, and at 3 dpi, respectively, and of IL-12, TNF-α, and IL-1β in serum at 1 and 3 dpi, at 5 and 7 dpi, and at 1 and 3 dpi, respectively. In addition, *C. butyricum* S-45-5-Cell led to a significant reduction in the amounts of IL-6, IL-12, TNF-α, and IL-1β in lung homogenates at 3, 5, and 7 dpi, at 1, 5, and 7 dpi, at 3 dpi, and at 1 and 3 dpi, respectively, and in the amounts of IL-6, IL-12, TNF-α, and IL-1β in serum at 3 dpi, at 1, 3, 5, and 7 dpi, at 5 and 7 dpi, and at 1 and 3 dpi, respectively. Moreover, *C. butyricum* S-45-5-Mix significantly reduced the amounts of IL-6, IL-12, TNF-α, and IL-1β in lung homogenates at 3, 5, and 7 dpi, at 1, 5, and 7 dpi, at 5 dpi, and at 1 dpi, respectively, and in the amounts of IL-6, IL-12, TNF-α, and IL-1β in serum at 3 and 5 dpi, at 3, 5, and 7 dpi, at 3, 5, and 7 dpi, and at 1 and 3 dpi, respectively (**Figures 5B, C**). These results indicate that inoculation with *C. butyricum* S-45-5 strain can protect mice from influenza infection that might be facilitated by downregulation of both local and systemic inflammatory responses, thereby preventing cytokine storm-mediated organ damage (particularly lung injury). Finally, we wanted to know whether *C. butyricum* S-45-5 strain regulates the secretion of well-known anti-inflammatory cytokines in influenza-virus-infected mice. As shown in **Figures 5B, C**, *C. butyricum* S-45-5-Cell and *C. butyricum* S-45-5-Sup induced secretion of IL-4 and IL-10 in lung homogenates and serum at 5 and 7 dpi. The levels of IL-4 and IL-10 induced in lung homogenates by *C. butyricum* S-45-5-Mix were significant at 7 dpi. Taken together, these results demonstrate that survival of *C. butyricum* S-45-5-treated mice after infection with pathogenic influenza virus is linked to increased secretion of anti-inflammatory cytokines, which prevents onset of a cytokine storm.

## Discussion

Probiotics can be described as “viable microorganisms that, when administered in sufficient quantities, provide a positive health effect to the host.” Even though some types of probiotics are targeted for animal use, most are used to promote human health by influencing the microbiome composition in the gut (33). Studies have shown that probiotics can successfully treat various respiratory, intestinal, and urogenital viral diseases (34–37). As viruses become more resistant to common antiviral agents, these antiviral probiotics could serve as a compelling substitute for traditional antiviral drugs (38). The basic mechanisms proposed as the basis for probiotic activity against viruses include direct interactions between probiotic cells and the specific targeting of viruses, the synthesis of antiviral metabolites, and the modulation of the eukaryotic host’s immune response (39, 40). Additionally, there exist possibilities for modulating the host’s microbiota, which engages with eukaryotic epithelial cells and exerts an impact on electrolyte potential (41).

Among the probiotics, *C. butyricum* is a symbiotic, spore-forming, butyrate-producing, obligate anaerobe Gram-positive bacteria that resides in a variety of environments. It can be found in the gastrointestinal tract of roughly 20% of the adult population (42). *C. butyricum* thrives through the fermentation of dietary fiber and other compounds resistant to digestion by the host’s enzymatic processes. The *but-buk* pathway is used during the fermentation of dietary fibers by *C. butyricum* (43). Representatively, *C. butyricum* MIYAIRI 588 (CBM 588) is well known in Japan as a treatment for gastrointestinal diseases such as diarrhea. *C. butyricum* MIYAIRI 588 has additionally been documented to modulate the expression of a range of cytokines, encompassing pro-inflammatory and anti-inflammatory cytokines (20). Specifically, it prompts the differentiation of T cells, specifically IL-17-producing (TJ) T cells, which play a vital role in promoting the expression of mucins and tight junction proteins within the colon’s epithelial cells (21). *C butyricum* MIYAIRI 588 also diminishes intestinal inflammation by modifying lipid metabolism through upregulating protectin-D1, an anti-inflammatory lipid mediator (20).

Here, we isolated a new strain of *Clostridium butyricum* from feces of newborn infant in Korea and designated as *C. butyricum* S-45-5. In particular, *C. butyricum* S-45-5 showed a different nucleotide sequence from *C. butyricum* MIYAIRI 588 and beneficial characteristics as an probiotic and exhibited potent antiviral effects both *in vitro* and *in vivo*. First, the suitability of *C. butyricum* S-45-5 as a potential probiotic bacterium was investigated by evaluating the physiological characteristic such as resistance to acid, bile acid, digestive enzymes, and their antagonistic effect on harmful bacteria, their antibiotic susceptibility, and their impact on growth of lactic acid bacteria. Second, we showed that *C. butyricum* S-45-5-Cell and *C. butyricum* S-45-5-Sup significantly reduced the replication of influenza (PR8-GFP), NDV-GFP, and HSV-GFP viruses in RAW246.7 murine macrophage cell line. Third, we demonstrated that *C. butyricum* S-45-5 exerts antiviral effects by activating innate immune responses and establishing an antiviral status in RAW246.7 cells. Fourth, oral administration of *C. butyricum* S-45-5 to BALB/c mice exhibited prophylactic effects against different influenza virus subtypes. These prophylactic effects are attributed to the induction of an antiviral state and immunomodulation to alleviate the local and systemic inflammatory responses caused by influenza virus challenge.

The development of probiotic strains involves many stages, including describing metabolic and physiological characteristics and evaluating biological properties (such as resistance to bile, gastric, and pancreatic secretions) (44, 45). In this study, the isolated *C. butyricum* S-45-5 showed typical characteristics of *C. butyricum*, including bile acid resistance, antibacterial ability, and growth promotion of various lactic acid bacteria for improving the profile of beneficial microorganisms. Moreover, the 1,424-bp 16S rRNA gene sequence result of *C. butyricum* S-45-5 identified *C. butyricum* VPI3266 (98.9%), *C. butyricum* LQ25 (98.8%), and *C. butyricum* JCM 1391^T^ (98.7%) as its close related strains; meanwhile, the utilization of the maximum-likelihood method, coupled with neighbor-joining and minimum-evolution analyses in phylogenetic investigations, indicated that this particular isolate signifies a novel strain within the *C. butyricum* species. The *C. butyricum* S-45-5 genome was 4,588,510 bp in size, with a DNA G+C content of 28.7 mol%, and closely related species within the species *C. butyricum* taxonomic group exhibited a ANI values ranged from 97.6% to 99.6%, and the dDDH values ranged from 78.8% to 96.3%. These results clearly indicate that the isolate represented a novel taxonomic entity within the broader category of the *C. butyricum* species.

The early induction of an antiviral state after virus infection is crucial to controlling the spread and pathogenic effects of viruses (46). Upon virus infection, host epithelial and immune cells recognize the virus and rapidly induce production of type I IFNs and cytokines to enhance antiviral responses through cellular defense mechanisms (47). For instance, TNF-α, which is important for immune cell activation (48); IL-12, which is important for T cell differentiation (49); IL-6, which is essential for differentiation of CD4^+^ T cells (50); IL-18 and TGF-β, which are responsible for promoting adaptive immune response, have been reported (51). Therefore, induction of IFNs and cytokines by specific stimulants could be an important approach to limit viral infection (52). A recent study reports that *Bacteroides fragilis* induces IFN-β expression through binding its capsular polysaccharide A to TLR4 receptors expressed by DCs in the colonic lamina propria, thereby protecting mice against VSV infection (53). Likewise, we hypothesized that *C. butyricum* S-45-5 induces an antiviral state by inducing the expression of IFN and pro-inflammatory cytokines. Based on our *in vitro* results, cells and culture supernatants of *C. butyricum* S-45-5 activated type I IFN and NF-κB signaling cascades in murine macrophage RAW264.7 cell line, leading to rapid production of IFN-β and pro-inflammatory cytokine IL-6 that play a crucial role in establishing a strong innate immune status. Secreted IFNs activate the JAK/STAT signaling cascade, leading to the initiation of hundreds of ISG gene transcription. As we have demonstrated in **Figure 2D**, transcription of IFNs and ISGs was significantly increased upon *C. butyricum* S-45-5 treatment. These ISGs might work alone or together to achieve one or several cellular results such as establishing antiviral defense or stimulation of adaptive immune response (31). Consequently, *C. butyricum* S-45-5 inhibited both RNA and DNA virus replication.

*In vivo*, we also demonstrated that oral administration of *C. butyricum* S-45-5 protects BALB/c mice against lethal infection by different influenza viruses. Oral administration of *C. butyricum* S-45-5 significantly reduced the virus titer in the lungs at 3 and 5 dpi, and pathological examination of the lungs showed that *C. butyricum* S-45-5 treatment in mice alleviates changes caused by influenza virus infection. This was demonstrated through the decrease in alveolar wall thickness and inflammatory cell infiltration into the alveoli. To support the antiviral effects of *C. butyricum* S-45-5 *in vivo*, when investigating the immune status of BALF, serum, and SIF from mice treated with *C. butyricum* S-45-5, we interestingly detected significant increases in IFN-γ, IFN-β, IL-12, and IL-6 in BALF, serum, and SIF. In addition, the lung expression of the antiviral and IFNs-related genes was enhanced in *C. butyricum* S-45-5-treated mice.

It is still unclear how orally administrated *C. butyricum* S-45-5 protects BALB/c mice from intranasally inoculated lethal influenza viruses. Among the several possible mechanisms, the effects of secreted IFNs and cytokines by the immunomodulatory properties of *C. butyricum* S-45-5 in the intestinal mucosa will have the greatest influence on respiratory antiviral immunity. However, it is necessary to conduct detailed studies to find out the exact mechanism of the effect on respiratory antiviral immunity. In addition, it is imperative to carry out studies on the immunomodulatory properties of *C. butyricum* S-45-5 by complex interactions between various molecules from *C. butyricum* S-45-5 and diverse immune or non-immune cells in the intestinal mucosa.

It is known that the major cause of lung injury and mortality after influenza virus infection is induced by virus replication and uncontrolled inflammatory responses, which is characterized by an excessive production of pro-inflammatory cytokines (32, 54, 55). This excessive production of pro-inflammatory cytokines is also induced by type I IFNs, which play a critical role in antiviral responses (32, 54, 55). Therefore, protection against influenza virus infection requires well-controlled production of IFNs, pro-inflammatory factors, and appropriate regulation with anti-inflammatory factors (32, 56). In this respect, we found that oral administration of *C. butyricum* S-45-5 reduced the production of several pro-inflammatory cytokines (IL-6, IL-12, IL-1β, and TNF-α) and significantly induced the secretion of anti-inflammatory cytokines (IL-4 and IL-10) in lung homogenates after challenge with influenza virus. These results suggest that *C. butyricum* S-45-5 may exert protective effects by downregulating excessive production of inflammatory cytokines after virus infection. Published evidence reports that probiotic proteins play a critical role in recognizing and activating immune cells. The significance of *Lactobacillus acidophilus* NCFM and a particular CD receptor known as DC-SIGN lies in their role in IL-12p70, IL-1β, and TNF-α release. Furthermore, when T cells are primed with *L. acidophilus* NCFM, they prompt DCs to discharge the IL-4 cytokine (57). Serine-threonine-rich proteins, referred to as STp in *Lactobacillus plantarum*, possess the ability to influence the phenotype of dendritic cells (DCs) in individuals with ulcerative colitis. Treating DCs with STp can lead to a decrease in TLR expression while enhancing the expression of CD40 and CD80 (58). The protease lactocepin secreted by *Lactobacillus paracasei* exhibits the capability to specifically break down the pro-inflammatory chemokine IFN-γ-inducible protein 10 (IP-10), which is involved in the recruitment of lymphocytes (59). However, the exact mechanism by which *C. butyricum* S-45-5 modulates the local and systemic inflammatory responses before and after influenza virus infection are still unknown and requires further investigation.

In conclusion, in this study, we isolated a new strain of *C. butyricum* S-45-5 and first evaluated its physiological characteristics as beneficial probiotics. Second, we demonstrated the antiviral properties of *C. butyricum* S-45-5 against influenza virus infection *in vitro* and *in vivo*. The mechanisms underlying the antiviral effect of *C. butyricum* S-45-5 involve induction of an early responses, including induction of type I IFNs, pro-inflammatory cytokines and ISGs, and modulation the balance between pro- and anti-inflammatory cytokines after virus infection. Therefore, *C. butyricum* S-45-5 may be used as a probiotic to alleviate or prevent influenza virus infection through immune modulation. Based on its mechanism, *C. butyricum* S-45-5 may also exhibit broad-spectrum antiviral effects against various respiratory or gastrointestinal viruses such as Norovirus, Rotavirus, and Astrovirus. Moreover, we wonder whether *C. butyricum* S-45-5 might exhibit beneficial effects in inflammatory diseases such as inflammatory bowel disease. However, further investigations are necessary to better understand the detailed immunomodulatory properties of *C. butyricum* S-45-5.

## Materials and methods

### Isolation of *C. butyricum* S-45-5, characterization, and culture conditions

The potential probiotic strain *C. butyricum* S-45-5, isolated from human feces, was selected by screening. To isolate anaerobic butyric-acid bacteria from adults and newborn infants, samples were heat-treated for 30 min at 80°C, plated on the Reinforced Clostridial Medium (RCM, Difco), and incubated under anaerobic conditions (anaerobic system, H_2_:CO_2_:N_2 = _8:5:88%, Forma Scientific) at 37°C for 48 h. The colonies that appeared after incubation were separated and kept at −80°C until use in the experiments. The isolated strains were identified by 16S rRNA gene analysis. To amplify 16S rDNA from purified isolates, polymerase chain reactions (PCRs) were carried out using forward primer 27F (5′-agagtttgatccctcag-3′) and a reverse primer 1492R (5′-ggttaccttgttacgactt-3′). The PCR conditions were as follows: 95°C for 2 min, followed by 30 cycles at 95°C for 20 s, at 50°C for 40 s, and at 72°C for 1 min, and the final cycle at 72°C for 5 min followed by 4°C for 10 min. The sizes of the amplified genes were first identified through gel electrophoresis, followed by sequencing analysis by Biofact Co. Ltd. The 16S rRNA gene sequence of the isolated specimen was aligned with sequences from prokaryotic type strains obtained from GenBank and EzBioCloud databases (www.ezbiocloud.net), which were previously recorded (60). The creation of phylogenetic trees was executed using unequivocal alignments. The alignment and subsequent phylogenetic evaluations were conducted using the PHYLIP and jPHYDIT software, respectively. To reconstruct phylogenetic trees, a combination of three algorithms, namely, neighbor joining, maximum likelihood, and minimum evolution, were utilized (61). A thorough assessment of the reliability of these phylogenetic trees was achieved via 1,000 bootstrap analyses. Additionally, the outgroup for these analyses was *Clostridioides difficile* ATCC 9689T.

The genomic DNA of the isolated specimen was readied and analyzed according to the techniques outlined by Shin et al. (62). Subsequently, the entire genomic library was sequenced and constructed on the Illumina platform, adhering to the protocol outlined by Macrogen Inc. based in Seoul, Korea. *De novo* assembly was undertaken using the RS HGAP Assembly (v3.0) with its default parameters, and error correction was conducted using Pilon (v1.21). Annotation of the assembled genome was accomplished using Prokka (v1.21b) (63). To gauge the genome’s completeness, the CheckM tool was employed. For the computation of the average nucleotide identity (ANI) of the genome, the ANI/AAI-Matrix calculator accessible at (http://enve-omics.ce.gatech.edu/g-matrix/index) was utilized. Furthermore, measurements of digital DNA–DNA hybridization (dDDH) were ascertained through the utilization of the Genome-to-Genome Distance Calculator (GGDC, version 3.0) using Formula 2 for calculation (64). Leveraging the autoMLST platform (https://automlst.ziemertlab.com), a multi-locus species tree predicated on 100 core genes inherent in the isolate’s entire genome sequences, and those of closely related strains, was formulated through IQ-TREE maximum-likelihood phylogeny (65). Furthermore, the whole genome sequences of these strains, accompanied by their respective GenBank accession numbers obtainable at (https://www.ncbi.nlm.nih.gov/genome), were harnessed for exhaustive scrutiny encompassing ANI, dDDH, and species phylogeny analyses.

Samples for the *in vitro* and *in vivo* experiments were prepared with newly cultured bacteria by titration of (*C. butyricum* S-45-5-Mix, which contains live bacteria and culture supernatant) or centrifuging at 4,000 rpm (4°C) for 20 min to separate cells from culture medium (*C. butyricum* S-45-5-Sup) and obtain live bacteria (*C. butyricum* S-45-5-Cell). To obtain *C. butyricum* S-45-5-Cell, after centrifuging, the culture supernatant was removed, and the bacteria pellet was washed three times using autoclaved PBS before resuspension in an appropriate amount of autoclaved PBS. Samples prepared for cell culture treatment were adjusted to pH 7.0. Samples prepared for the *in vivo* experiments were used directly, without pH adjustment.

### Cells and viruses

RAW264.7 (ATCC^®^ TIB-71™), Vero (ATCC^®^ CCL-81™), and MDCK (ATCC CCL-34, NBL-2) cell lines were cultured in Dulbecco’s modified Eagle’s medium (DMEM) (Cytiva), supplemented with 10% fetal bovine serum (FBS) (Gibco) and 1% antibiotic-antimycotic solution (Gibco). These cell lines were maintained at a temperature of 37°C in a 5% CO_2_ environment. Green Fluorescent Protein (GFP)-fused Influenza A PR8-GFP (A/Puerto Rico/8/1934), Newcastle Disease Virus (NDV-GFP) and challenge viruses of influenza A subtypes H1N1 (A/Puerto Rico/8/1934), H3N2 (A/Philippines/2/2008), and H9N2 (A/Chicken/Korea/116/2004) were propagated in the allantoic fluid of 10-day-old chicken embryos. Herpes Simplex Virus (HSV-GFP) was propagated on confluent Vero cells.

### Virus titration assay

Vero cells were used to assess the viral titers through the standard plaque assay method (66) for PR8-GFP and HSV-GFP with some modifications. To assay the viral replication in PR8-GFP and HSV-GFP infected cells, culture supernatants and cells were collected, and cell pellets were subjected to three cycles of freezing at −70°C and thawing at room temperature, and titer was determined in Vero cells using standard plaque assay. To determine the NDV-GFP virus titer, complete RNAs were extracted from RAW264.7 cells infected with NDV-GFP using RNeasy Mini Kit (Qiagen), and to synthesize cDNA, reverse transcription was performed using reverse transcriptase (Toyobo, Japan) following manufacturer’s instruction. To determine NDV-GFP mRNA expression levels, real-time polymerase chain reaction (RT-PCR) was performed using gene-specific primers. NDV Matrix (M) gene, forward primer was 5′-TCGAGICTGTACAATCTTGC-3′ and the reverse primer was 5′- GTCCGAGCACATCACTGAGC-3′.

### Trypan blue exclusion assay

The trypan blue exclusion assay was employed to assess cell viability following *C. butyricum* S-45-5 treatment. RAW264.7 cells and HEK293T cells were initially seeded in separate 12-well cell culture plates and allowed to attach for 12 h. After this period, the cells were exposed to *C. butyricum* S-45-5. At 24 h post-treatment, the cells were collected and mixed with a 0.4% trypan blue solution that was diluted 1:1 with cell culture medium. The cell–dye mixture was incubated for 3–5 min to allow for dye penetration into non-viable cells. Subsequently, stained cells were loaded onto a hemocytometer chamber, and viable and non-viable cells were counted under a light microscope. Viability was calculated by dividing the number of viable cells by the total cell count and multiplying by 100.

### Detection of IFN-β and pro-inflammatory cytokines

RAW264.7 cells were treated with PBS (medium), 100 U/ml rmIFN-β, or *C. butyricum* S-45-5-Cell (1 × 10^6^ CFU/ml) or S-45-5-Sup (1 × 10^6^ CFU/ml). Collected cell supernatants at 12 hpt and 24 hpt were centrifuged at 2,500×*g* for 10 min at 4°C for clarification and used for the subsequent ELISA assays. Mouse IFN-β (PBL Interferon Source, USA) or mouse IL-6 (BD Bioscience, USA) kits were used. All the steps were followed as indicated in the manufacturer’s protocol instructions.

### Immunoblot analysis

RAW264.7 cells were seeded into TC-treated six-well tissue culture plates (1 × 10^6^ cells/well) and incubated at 37°C under 5% CO_2_. Twelve hours later, cells were treated with DMEM media alone (negative control), DMEM with 100 ng/ml LPS (positive control), or DMEM with *C. butyricum* S-45-5-Cell (1 × 10^6^ CFU/ml) or S-45-5-Sup (1 × 10^6^ CFU/ml), and the cells were collected at 0, 8, 12, and 16 hpt. After washing the pellets with PBS, whole cell lysates were subjected to SDS-PAGE followed by immunoblotting with antibodies indicated: anti-IRF3 (Cell Signaling, 4302S), anti-phospho-IRF3 (Ser396) (Cell Signaling, 4947S), anti-STAT1 (Cell Signaling, 9172), anti-phospho-STAT1 (Tyr701) (Cell Signaling, 9167S), anti-p65 (Cell Signaling, 4764S), anti-phospho-p65 (Cell Signaling, 3031S), anti-IkBα (Cell Signaling, 9242S), anti-phospho-IkBα (Cell Signaling, 2859S), or anti-β-actin (Santa Cruz, SC#47778) antibodies. A Las-3000 miniLumino Image Analyzer (ECL-GE Healthcare, UK) equipped with a Chemiluminescence Detection System (ECL-GE Healthcare, UK) was used to visualize the respective proteins using a horseradish peroxidase-conjugated secondary antibody (Sigma, USA).

### mRNA quantification by real-time polymerase chain reaction

RAW264.7 cells were seeded in six-well tissue culture plates with the cell number of 1 × 10^6^ cells/well and incubated at 37°C with 5% CO_2_ condition. The cells were treated with DMEM alone (negative control) and DMEM with *C. butyricum* S-45-5-Cell (1 × 10^6^ CFU/ml) or *C. butyricum* S-45-5-Sup (1 × 10^6^ CFU/ml), and the cells were harvested at 24 hpt. RNeasy Mini Kit (Qiagen, Seoul, Korea) was used to isolate total RNA from harvested cells, and reverse transcriptase (Toyobo, Japan) was used to synthesize cDNA. Quantification of cDNA levels was carried out by quantitative polymerase chain reaction (qPCR) using a QuantiTect SYBR Green PCR kit (Qiagen, Korea) on a PCR Thermal Cycler Dice, model No. TP600 (TaKaRa, Japan) with the PCR primers listed in **Supplementary Table S3**.

### *In vivo* challenge test in BALB/c mice

*In vivo* trials were executed employing female BALB/c mice, aged 6 weeks (**Supplementary Table S4**). To conduct survival rate analysis, a total of 96 mice were divided into three primary groups depending on the challenge virus that they were exposed to (H1N1, H3N2, and H9N2), and each group was divided into three subgroups as non-infected (PBS, n=6), virus only (negative control, n=6), mrIFN-β (positive control n=6), *C. butyricum* S-45-5-Mix (consist of live bacteria and culture supernatant; 5 × 10^7^ CFU, n=6), *C. butyricum* S-45-5-Cell (5 × 10^7^ CFU, n=6), or *C. butyricum* S-45-5-Sup (5 × 10^7^ CFU, n=6). For the lung virus (H1N1 infected only) titration, another 32 mice were grouped into four sub-groups as non-infected (PBS, n=2), virus only (negative control, n=6), mrIFN-β (positive control n=6), *C. butyricum* S-45-5-Mix (5 × 10^7^ CFU, n=6), *C. butyricum* S-45-5-Cell (5×10^7^ CFU, n=6), or *C. butyricum* S-45-5-Sup (5 × 10^7^ CFU, n=6). The mice were orally administered *C. butyricum* S-45-5 at a total volume of 100 μl (5 × 10^7^ CFU/ml) and for the control group, 100 μl of PBS using oral gavage (22-gauge/5 cm long) for 21 days. mrIFN-β was treated intranasally before 12 h of infection as a positive control. Before the *in vivo* influenza virus challenge, 50% mouse lethal dose (MLD_50_) was determined for each influenza subtype in BALB/c mice. Except for non-infected groups, all other mice were intranasally infected (30 μl) with the 2MLD_50_ of H1N1 (A/PR/8/34), H3N2 (A/Philippines/2/2008), and H9N2 (A/Chicken/Korea/116/2004). Following the infection, mice were monitored continuously for 13 days, and the weight loss and survival were measured at fixed time points each day. Mice in the lung virus titration group were randomly sacrificed 3 (n=3) and 5 dpi (n=3) to measure the lung virus titers. Lung tissues were kept at –70°C deep freezer until further use for the virus titration (left lobe of the lung was used for the virus titration). During the sample collection at 5 dpi, the left lobe of the lung was immediately fixed in 10% formalin for histopathology analysis. The survival rate was determined by death or body weight loss, which is cut off at a 25% reduction. Following the conclusion of the last monitoring session, all mice that had survived were subjected to a humane euthanasia process involving a 5-min exposure to CO_2_ inhalation.

### Determination of lung virus titer and histopathology

The virus titers in the lungs were assessed as previously demonstrated using 50% tissue culture infectious dose (TCID_50_) assay (67). Briefly, to remove cellular debris, the lung tissues were centrifuged (12,000 rpm for 15 min.) after being homogenized in PBS containing antibiotic and antimycotic solution (Gibco, USA). Serially diluted samples were incubated with confluent MDCK cells in 96-well cell culture plates at 37°C in a 5% CO_2_ atmosphere for 1 h, and overlay medium containing L-1-tosylamide-2-phenylethyl chloromethyl ketone-(TPCK-) trypsin (Sigma-Aldrich) was replaced with DMEM that does not include FBS. Then, infected cells were incubated for 3–4 days. The cytopathic effect (CPE) was incubation with a microscope, and virus titers were calculated by the Reed and Muench method and expressed as log_10_ TCID_50_/lung tissue (68). For the histopathology sample preparation, tissues were preserved in 10% formalin-containing neutral buffer right after collection, and samples were kept for 2 days on the shaker. Next, samples were embedded in paraffin wax using the middle part of the tissues. The 4–6-mm-thick sections were prepared by using a microtome, mounted on slides, and stained with hematoxylin and eosin (H&E). Light microscopy was used to assess histopathological changes, as previously described (69, 70).

### Evaluation of cytokines and RT-PCR of lung tissue

A total of 20, 6-week-old female BALB/c mice were randomly divided into five groups (n=4). Mice were orally administrated with *C. butyricum* S-45-5-Mix (5 × 10^7^ CFU), *C. butyricum* S-45-5-Cell (5 × 10^7^ CFU), or *C. butyricum* S-45-5-Sup (5 × 10^7^ CFU) in a total volume of 100 µl and control mice with 100 µl of PBS daily for 21 days as separate groups. mrIFN-β was treated intranasally prior to 12 h of sample collection as a positive control. On the following day, several samples were gathered, including serum, bronchoalveolar lavage fluid (BALF), and small-intestinal fluid (SIF). Collected tissue/samples were stored at −70°C for subsequent analysis. The serum was collected via the retro-orbital plexus, centrifuged, and stored at −70°C for subsequent ELISA analysis (murine IFN-β, IFN-γ, IL-6, and IL-12). For BALF, it was collected as discussed before with some modifications. Hank’s Balanced Salt Solution (HBSS) was used to lavage the mice lungs four times, which were then collected, stored at −70°C, and later subjected to an ELISA test (mouse IFN-γ, IFN-β, IL-12, and IL-6). After obtaining BALF, we proceeded to gather lung tissue specimens for the purpose of conducting RT-PCR and stored at −70°C until analysis. For SIF, an incision along the midline and subsequent skin retraction were executed subsequent to the euthanized mice through cervical dislocation. The small intestine was cut approximately 1 cm above the cecum to separate it from others. Following a meticulous 500 ml HBSS (Sigma-Aldrich) flush of the intestine, the collected fluids underwent centrifugation and were then preserved at a temperature of −20°C. The SIF supernatants were further assayed by the ELISA (mouse IFN-γ, IFN-β, IL-12, and IL-6),.

### Determination of serum and lung cytokine levels in *C. butyricum* S-45-5-treated mice upon H1N1 infection

A total of 52, 6-week-old female BALB/c mice were randomly allocated into five groups as non-infected (PBS, n=4), virus only (negative control, n=12), *C. butyricum* S-45-5-Mix (5 × 10^7^ CFU, n=12), *C. butyricum* S-45-5-Cell (5 × 10^7^ CFU, n=12), or *C. butyricum* S-45-5-Sup (5 × 10^7^ CFU, n=120. The mice were orally administered *C. butyricum* at a total volume of 100 μl and for the control group, 100 μl of PBS for 21 consecutive days. All mice except the non-infected group were anesthetized and intranasally infected (30 μl) with the 50% mouse lethal dose (1MLD_50_) of H1N1 on infection day (day 0). A subset of mice (n=13) was sacrificed on 1 dpi, and serum and lung tissues were collected. Similarly, samples were collected at 3, 5, and 7 dpi. Pro-inflammatory (IL-6, IL-12, TNF-α, and IL-1β) and anti-inflammatory cytokine levels (IL-4, IL-10) were measured with ELISA according to the manufacturer’s instructions.

### Acid resistance, bile acid resistance, and digestive enzyme resistance tests

In the acid resistance test, *C. butyricum* S-45-5 was inoculated into RCM liquid media where pH was adjusted to 1.0–6.0, respectively, using HCl and incubated at 37°C for 48 h. The survival rates were measured using optical densities (OD 600). In the bile acid resistance test, Bacto Oxgall (Difco) at concentrations of 0.3, 1.0, and 3.0% (w/v) were added to the RCM plated medium, respectively, to culture strain of *C. butyricum* S-45-5, and the growth rate was measured by plate count method. To measure the ability to impede digestive enzymes such as amylase, cellulase, lipase, and protease, respective analytical reagents were added to the RCM solid medium. The bacteria were cultured for at least 3 days, and clear zones were identified using dyeing reagents (71).

### Pathogen antagonistic ability test

Harmful bacteria mainly related to intestinal diseases consisting of 12 species. *E. coli* (KCTC 2441), *Klebsiella pneumoniae* (KCTC 2208), *Shigella flexneri* (KCTC 22192), *Enterobacter cloacea* (KCTC 1685), *Pseudomonas aeruginosa* (KCTC 2004), *Vibrio parahaemolyticus* (KCTC 2729), *Staphylococcus aureus* (KCTC 3881), *Enterobacter aerogenes* (KCTC 2190), *Clostridium difficile* (KCTC 5009), *Campylobacter jejuni* (KCTC 5327), *Clostridium perfringens* (KCTC 3269), and *Fusobacterium varium* (KCTC 15085) purchased from the Korea Collection for Type Culture (KCTC) of the Korea Research Institute of Bioscience and Biotechnology and three species, *E. coli* O157, *Salmonella typhimurium*, and *Salmonella enteritidis* purchased from Korea Centers for Disease Control and Prevention were used as indicator strains. After adjusting the concentrations of the indicator strains cultured for approximately 24 h to 10^6^ CFU/ml, the indicator strains were applied to Mueller–Hinton agar medium and dried. After that, 100 μl of the culture supernatant of the isolated strain was inoculated into the sterilized disk and incubated for 48 h. The supernatant was prepared by adjusting the pH of the liquid culture medium to pH 4 and pH 6 and removing the microbial cells with membrane filters (0.22 μl pore size, Sartorius, France). The pH-adjusted RCM liquid medium blank was used as a control in this assay. The diameters (mm) of the inhibition rings were measured using paper disks (71).

### Analysis of organic acid metabolites

Organic acid metabolites were analyzed using gas chromatography (GC). The culture supernatant obtained by incubating isolates in the PYG liquid medium at 37°C for 48 h. Ethanol, acetic acid, butanol, and butyric acid were used as standards for the analysis. The culture supernatant and standards were filtered with 0.22-μm filters, mixed with an equal volume of 10% (v/v) phosphoric acid, injected into a GC-FID installed with a HP-INNOWax column (60 m × 250 μm × 0.25 μm, Agilent Technologies) for analysis. Employing helium as the carrier gas, the flowrate was set at 1 ml/min. Concurrently, the oven temperature was gradually raised from 50°C to 170°C; it was programmed to increase by 10°C/min. The injector and detector temperatures were set to 250°C (72).

### Antibiotics susceptibility test

A total of nine antibiotics, kanamycin (30µg), penicillin (10µg), cephalothin (30 µg), clindamycin (2µg), tetracycline (30 µg), gentamycin (10µg), streptomycin (10 µg), ampicillin (10 µg), and vancomycin (30 µg) were used. In the test, the isolated strain (10^6^ CFU/ml) was applied to the Muller–Hinton medium, and antibiotic disks were inoculated on the medium. After incubation at 37°C for 48 h, the diameters (mm) of the inhibition rings were measured, and tolerance was determined according to the sizes (72).

### Lactic acid bacteria proliferation promotion test

To carry out lactic acid bacteria growth promotion tests, *Lactobacillus* and *Bifidobacterium* were used as follows: *L. plantarum* (KCTC 3108), *Lactobacillus reuteri* (KCTC 3564), *Lactobacillus salivarius* (KCTC 3600), *L. rhamnosus* (KCTC 3237), *L. paracasei* subsp. *paracasei* (KCTC 3510), *L. sakei* subsp. *sakei* (KCTC 3603), *Bifidobacterium longum* subsp. *longum* (KCTC 3128), and *Bifidobacterium catenulatum* (KCTC 3221). The tests were prepared as described elsewhere (73) with some modifications. The lactic acid bacteria proliferation was tested using both culture solution and supernatant of the *C. butyricum* S-45-5. The culture solution of the *C. butyricum* S-45-5 and lactic acid bacteria were mixed at a ratio of 1:1 (v/v). Then, 10 ml of fresh MRS liquid medium was seeded with the 2% mixture and cultured 1 d at 37°C. The CFU/ml of lactic acid bacteria was analyzed after the 50 ml culture was spread on the MRS plate and cultured 1 d at 37°C. The mixture without the culture solution of *C. butyricum* S-45-5 was used as a control. The test of the supernatant was carried out with the same method by using the supernatant of *C. butyricum* S-45-5 instead of the culture solution.

### Statistical analysis

Data are represented as the means ± standard deviations (SD) of results from three independent experiments with similar patterns. Graphs and all statistical analysis were performed using GraphPad Prism software version 6 (San Diego, USA) for Windows. Differences between the means were compared using Student’s t-test. In all experiments, *p<0.05, **p<0.01, and ***p<0.001 were regarded as differentially significant, significant, and highly significant, respectively. The survival times were compared with log-rank tests using GraphPad Prism 6.0.

## Data availability statement

The datasets presented in this study can be found in online repositories. The names of the repository/repositories and accession number(s) can be found below: https://www.ncbi.nlm.nih.gov/, GCA_003315755.1.

## Ethics statement

The studies involving humans were approved by Medical Ethics Committee of Inje University College of Medicine (13–151). The studies were conducted in accordance with the local legislation and institutional requirements. Written informed consent for participation in this study was provided by the participants’ legal guardians/next of kin. The animal study was approved by Chungnam National University Institutional Animal Care and Use Committee (Reference numbers CNU-00509, CNU-767, and 202203A-CNU-027). The study was conducted in accordance with the local legislation and institutional requirements.

## Author contributions

KC, YSS, and MBU performed the experiments. JP, WC, YBS, and LB helped with the experiments. HK, JS contributed to the discussions. Y-HC and J-SL designed the study. KC, MBU, Y-HC, and JSL wrote the manuscript. Y-HC and J-SL supervised the study. All the authors helped with data analysis. All authors contributed to the article and approved the submitted version.

## References

[1] Yoo DG Kim MC Park MK Park KM Quan FS Song JM. Protective effect of ginseng polysaccharides on influenza viral infection. PloS One (2012) 7(3):e33678. doi: 10.1371/journal.pone.0033678 22442708 PMC3307756

[2] Meganck RM Baric RS. Developing therapeutic approaches for twenty-first-century emerging infectious viral diseases. Nat Med (2021) 27(3):401–10. doi: 10.1038/s41591-021-01282-0 33723456

[3] Nellore A Fishman J. Pandemic swine flu 2009. Xenotransplantation (2009) 16(6):463–5. doi: 10.1111/j.1399-3089.2009.00559.x 20042043

[4] Zelaya H Alvarez S Kitazawa H Villena J. Respiratory antiviral immunity and immunobiotics: beneficial effects on inflammation-coagulation interaction during influenza virus infection. Front Immunol (2016) 7:633. doi: 10.3389/fimmu.2016.00633 28066442 PMC5179578

[5] Wang Y Moon A Huang J Sun Y Qiu HJ. Antiviral effects and underlying mechanisms of probiotics as promising antivirals. Front Cell infection Microbiol (2022) 12:928050. doi: 10.3389/fcimb.2022.928050 PMC9207339 35734576

[6] Abt MC Osborne LC Monticelli LA Doering TA Alenghat T Sonnenberg GF. Commensal bacteria calibrate the activation threshold of innate antiviral immunity. Immunity (2012) 37(1):158–70. doi: 10.1016/j.immuni.2012.04.011 PMC3679670 22705104

[7] Mudroňová D Karaffová V Csank T Király J Revajová V Gancarčíková S. Systemic immune response of gnotobiotic mice infected with porcine circovirus type 2 after administration of lactobacillus reuteri L26 biocenol™. Beneficial Microbes (2018) 9(6):951–61. doi: 10.3920/bm2017.0147 30232907

[8] Dimitrijevic R Ivanovic N Mathiesen G Petrusic V Zivkovic I Djordjevic B. Effects of lactobacillus rhamnosus la68 on the immune system of C57bl/6 mice upon oral administration. J dairy Res (2014) 81(2):202–7. doi: 10.1017/s0022029914000028 24559976

[9] Kawahara T Takahashi T Oishi K Tanaka H Masuda M Takahashi S. Consecutive oral administration of bifidobacterium longum mm-2 improves the defense system against influenza virus infection by enhancing natural killer cell activity in a murine model. Microbiol Immunol (2015) 59(1):1–12. doi: 10.1111/1348-0421.12210 25400245

[10] Molina MA Díaz AM Hesse C Ginter W Gentilini MV Nuñez GG. Immunostimulatory effects triggered by enterococcus faecalis cect7121 probiotic strain involve activation of dendritic cells and interferon-gamma production. PloS One (2015) 10(5):e0127262. doi: 10.1371/journal.pone.0127262 25978357 PMC4433276

[11] Park MK Ngo V Kwon YM Lee YT Yoo S Cho YH. Lactobacillus plantarum dk119 as a probiotic confers protection against influenza virus by modulating innate immunity. PloS One (2013) 8(10):e75368. doi: 10.1371/journal.pone.0075368 24124485 PMC3790790

[12] Starosila D Rybalko S Varbanetz L Ivanskaya N Sorokulova I. Anti-influenza activity of a bacillus subtilis probiotic strain. Antimicrobial Agents chemother (2017) 61(7). doi: 10.1128/aac.00539-17 PMC5487639 28416546

[13] Garcia-Castillo V Tomokiyo M Raya Tonetti F Islam MA Takahashi H Kitazawa H. Alveolar macrophages are key players in the modulation of the respiratory antiviral immunity induced by orally administered lacticaseibacillus rhamnosus crl1505. Front Immunol (2020) 11:568636. doi: 10.3389/fimmu.2020.568636 33133080 PMC7550464

[14] Eguchi K Fujitani N Nakagawa H Miyazaki T. Prevention of respiratory syncytial virus infection with probiotic lactic acid bacterium lactobacillus gasseri sbt2055. Sci Rep (2019) 9(1):4812. doi: 10.1038/s41598-019-39602-7 30886158 PMC6423325

[15] Yang CM Cao GT Ferket PR Liu TT Zhou L Zhang L. Effects of probiotic, clostridium butyricum, on growth performance, immune function, and cecal microflora in broiler chickens. Poultry Sci (2012) 91(9):2121–9. doi: 10.3382/ps.2011-02131 22912445

[16] Cross ML. Immune-signalling by orally-delivered probiotic bacteria: effects on common mucosal immunoresponses and protection at distal mucosal sites. Int J immunopathol Pharmacol (2004) 17(2):127–34. doi: 10.1177/039463200401700204 15171813

[17] Cassir N Benamar S La Scola B. Clostridium butyricum: from beneficial to a new emerging pathogen. Clin Microbiol infection (2016) 22(1):37–45. doi: 10.1016/j.cmi.2015.10.014 26493849

[18] Samuelson DR Welsh DA Shellito JE. Regulation of lung immunity and host defense by the intestinal microbiota. Front Microbiol (2015) 6:1085. doi: 10.3389/fmicb.2015.01085 26500629 PMC4595839

[19] Murayama T Mita N Tanaka M Kitajo T Asano T Mizuochi K. Effects of orally administered clostridium butyricum miyairi 588 on mucosal immunity in mice. Veterinary Immunol immunopathol (1995) 48(3-4):333–42. doi: 10.1016/0165-2427(95)05437-b 8578691

[20] Ariyoshi T Hagihara M Eguchi S Fukuda A Iwasaki K Oka K. Clostridium butyricum miyairi 588-induced protectin D1 has an anti-inflammatory effect on antibiotic-induced intestinal disorder. Front Microbiol (2020) 11:587725. doi: 10.3389/fmicb.2020.587725 33193245 PMC7661741

[21] Hagihara M Kuroki Y Ariyoshi T Higashi S Fukuda K Yamashita R. Clostridium butyricum modulates the microbiome to protect intestinal barrier function in mice with antibiotic-induced dysbiosis. iScience (2020) 23(1):100772. doi: 10.1016/j.isci.2019.100772 31954979 PMC6970176

[22] Hayashi A Sato T Kamada N Mikami Y Matsuoka K Hisamatsu T. A single strain of clostridium butyricum induces intestinal il-10-producing macrophages to suppress acute experimental colitis in mice. Cell Host Microbe (2013) 13(6):711–22. doi: 10.1016/j.chom.2013.05.013 23768495

[23] Sokol H Pigneur B Watterlot L Lakhdari O Bermúdez-Humarán LG Gratadoux JJ. Faecalibacterium prausnitzii is an anti-inflammatory commensal bacterium identified by gut microbiota analysis of crohn disease patients. Proc Natl Acad Sci United States America (2008) 105(43):16731–6. doi: 10.1073/pnas.0804812105 PMC2575488 18936492

[24] Parks DH MacDonald NJ Beiko RG. Classifying short genomic fragments from novel lineages using composition and homology. BMC Bioinf (2011) 12:328. doi: 10.1186/1471-2105-12-328 PMC3173459 21827705

[25] Fontana L Bermudez-Brito M Plaza-Diaz J Munoz-Quezada S Gil A. Sources, isolation, characterisation and evaluation of probiotics. Br J Nutr (2013) 109(S2):S35–50. doi: 10.1017/S0007114512004011 23360880

[26] Gunn JS. Mechanisms of bacterial resistance and response to bile. Microbes infection (2000) 2(8):907–13. doi: 10.1016/s1286-4579(00)00392-0 10962274

[27] Atasoy M Cetecioglu Z. Butyric acid dominant volatile fatty acids production: bio-augmentation of mixed culture fermentation by clostridium butyricum. J Environ Chem Eng (2020) 8(6):104496. doi: 10.1016/j.jece.2020.104496

[28] Kiso M Takano R Sakabe S Katsura H Shinya K Uraki R. Protective efficacy of orally administered, heat-killed lactobacillus pentosus B240 against influenza a virus. Sci Rep (2013) 3:1563. doi: 10.1038/srep01563 23535544 PMC3610098

[29] Thangavel RR Bouvier NM. Animal models for influenza virus pathogenesis, transmission, and immunology. J Immunol Methods (2014) 410:60–79. doi: 10.1016/j.jim.2014.03.023 24709389 PMC4163064

[30] Shaw AE Hughes J Gu Q Behdenna A Singer JB Dennis T. Fundamental properties of the mammalian innate immune system revealed by multispecies comparison of type I interferon responses. PLoS Biol (2017) 15(12):e2004086. doi: 10.1371/journal.pbio.2004086 29253856 PMC5747502

[31] Schoggins JW. Interferon-stimulated genes: what do they all do? Annu Rev Virol (2019) 6(1):567–84. doi: 10.1146/annurev-virology-092818-015756 31283436

[32] Liu Q Zhou YH Yang ZQ. The cytokine storm of severe influenza and development of immunomodulatory therapy. Cell Mol Immunol (2016) 13(1):3–10. doi: 10.1038/cmi.2015.74 26189369 PMC4711683

[33] Hill C Guarner F Reid G Gibson GR Merenstein DJ Pot B. Expert consensus document. The international scientific association for probiotics and prebiotics consensus statement on the scope and appropriate use of the term probiotic. Nat Rev Gastroenterol Hepatol (2014) 11(8):506–14. doi: 10.1038/nrgastro.2014.66 24912386

[34] Nader-Macías MEF De Gregorio PR Silva JA. Probiotic lactobacilli in formulas and hygiene products for the health of the urogenital tract. Pharmacol Res Perspect (2021) 9(5):e00787. doi: 10.1002/prp2.787 34609059 PMC8491456

[35] Yan F Polk DB. Probiotics and probiotic-derived functional factors—Mechanistic insights into applications for intestinal homeostasis. Front Immunol (2020) 11:1428. doi: 10.3389/fimmu.2020.01428 32719681 PMC7348054

[36] Zhao Z Xu S Zhang W Wu D Yang G. Probiotic escherichia coli nissle 1917 for inflammatory bowel disease applications. Food Funct (2022) 13(11):5914–24. doi: 10.1039/D2FO00226D 35583304

[37] Jamalkandi SA Ahmadi A Ahrari I Salimian J Karimi M Ghanei M. Oral and nasal probiotic administration for the prevention and alleviation of allergic diseases, asthma and chronic obstructive pulmonary disease. Nutr Res Rev (2021) 34(1):1–16. doi: 10.1017/S0954422420000116 32281536

[38] Dutkowski R. Oseltamivir in seasonal influenza: cumulative experience in low- and high-risk patients. J antimicrobial chemother (2010) 65 Suppl 2(Suppl 2):ii11–24. doi: 10.1093/jac/dkq012 PMC2835508 20215131

[39] Yitbarek A Alkie T Taha-Abdelaziz K Astill J Rodriguez-Lecompte JC Parkinson J. Gut microbiota modulates type I interferon and antibody-mediated immune responses in chickens infected with influenza virus subtype H9n2. Beneficial Microbes (2018) 9(3):417–27. doi: 10.3920/bm2017.0088 29380643

[40] Al Kassaa I Hober D Hamze M Chihib NE Drider D. Antiviral potential of lactic acid bacteria and their bacteriocins. Probiotics antimicrobial Proteins (2014) 6(3-4):177–85. doi: 10.1007/s12602-014-9162-6 24880436

[41] Olaya Galán NN Ulloa Rubiano JC Velez Reyes FA Fernandez Duarte KP Salas Cárdenas SP Gutierrez Fernandez MF. *In vitro* antiviral activity of lactobacillus casei and bifidobacterium adolescentis against rotavirus infection monitored by nsp4 protein production. J Appl Microbiol (2016) 120(4):1041–51. doi: 10.1111/jam.13069 26801008

[42] Rolfe RD. Interactions among microorganisms of the indigenous intestinal flora and their influence on the host. Rev Infect Dis (1984) 6 Suppl 1:S73–9. doi: 10.1093/clinids/6.supplement_1.s73 6372040

[43] Vital M Penton CR Wang Q Young VB Antonopoulos DA Sogin ML. A gene-targeted approach to investigate the intestinal butyrate-producing bacterial community. Microbiome (2013) 1(1):8. doi: 10.1186/2049-2618-1-8 24451334 PMC4126176

[44] Guarner F Malagelada JR. Gut flora in health and disease. Lancet (London England) (2003) 361(9356):512–9. doi: 10.1016/s0140-6736(03)12489-0 12583961

[45] Saarela M Mogensen G Fondén R Mättö J Mattila-Sandholm T. Probiotic bacteria: safety, functional and technological properties. J Biotechnol (2000) 84(3):197–215. doi: 10.1016/s0168-1656(00)00375-8 11164262

[46] Boasso A. Type I interferon at the interface of antiviral immunity and immune regulation: the curious case of hiv-1. Scientifica (2013) 2013:580968. doi: 10.1155/2013/580968 24455433 PMC3885208

[47] Carty M Guy C Bowie AG. Detection of viral infections by innate immunity. Biochem Pharmacol (2021) 183:114316. doi: 10.1016/j.bcp.2020.114316 33152343

[48] Wang T He C. Tnf-α and il-6: the link between immune and bone system. Curr Drug Targets (2020) 21(3):213–27. doi: 10.2174/1389450120666190821161259 31433756

[49] Romee R Schneider SE Leong JW Chase JM Keppel CR Sullivan RP. Cytokine activation induces human memory-like nk cells. Blood (2012) 120(24):4751–60. doi: 10.1182/blood-2012-04-419283 PMC3520618 22983442

[50] Espinosa V Rivera A. Cytokines and the regulation of fungus-specific cd4 T cell differentiation. Cytokine (2012) 58(1):100–6. doi: 10.1016/j.cyto.2011.11.005 PMC3290683 22133343

[51] Smith AJ Humphries SE. Cytokine and cytokine receptor gene polymorphisms and their functionality. Cytokine Growth factor Rev (2009) 20(1):43–59. doi: 10.1016/j.cytogfr.2008.11.006 19038572

[52] Zhao J Wohlford-Lenane C Zhao J Fleming E Lane TE McCray PB Jr.. Intranasal treatment with poly(I•C) protects aged mice from lethal respiratory virus infections. J Virol (2012) 86(21):11416–24. doi: 10.1128/jvi.01410-12 PMC3486278 22915814

[53] Stefan KL Kim MV Iwasaki A Kasper DL. Commensal microbiota modulation of natural resistance to virus infection. Cell (2020) 183(5):1312–24.e10. doi: 10.1016/j.cell.2020.10.047 33212011 PMC7799371

[54] Walsh KB Teijaro JR Wilker PR Jatzek A Fremgen DM Das SC. Suppression of cytokine storm with a sphingosine analog provides protection against pathogenic influenza virus. Proc Natl Acad Sci United States America (2011) 108(29):12018–23. doi: 10.1073/pnas.1107024108 PMC3142000 21715659

[55] Tisoncik JR Korth MJ Simmons CP Farrar J Martin TR Katze MG. Into the eye of the cytokine storm. Microbiol Mol Biol Rev MMBR (2012) 76(1):16–32. doi: 10.1128/mmbr.05015-11 22390970 PMC3294426

[56] Gu Y Zuo X Zhang S Ouyang Z Jiang S Wang F. The mechanism behind influenza virus cytokine storm. Viruses (2021) 13(7):1362. doi: 10.3390/v13071362 34372568 PMC8310017

[57] Konstantinov SR Smidt H de Vos WM Bruijns SC Singh SK Valence F. S layer protein a of lactobacillus acidophilus ncfm regulates immature dendritic cell and T cell functions. Proc Natl Acad Sci United States America (2008) 105(49):19474–9. doi: 10.1073/pnas.0810305105 PMC2592362 19047644

[58] Al-Hassi HO Mann ER Sanchez B English NR Peake ST Landy J. Altered human gut dendritic cell properties in ulcerative colitis are reversed by lactobacillus plantarum extracellular encrypted peptide stp. Mol Nutr Food Res (2014) 58(5):1132–43. doi: 10.1002/mnfr.201300596 24347371

[59] von Schillde MA Hörmannsperger G Weiher M Alpert CA Hahne H Bäuerl C. Lactocepin secreted by lactobacillus exerts anti-inflammatory effects by selectively degrading proinflammatory chemokines. Cell Host Microbe (2012) 11(4):387–96. doi: 10.1016/j.chom.2012.02.006 22520466

[60] Chun J Goodfellow M. A phylogenetic analysis of the genus nocardia with 16s rrna gene sequences. Int J systematic bacteriol (1995) 45(2):240–5. doi: 10.1099/00207713-45-2-240 7537058

[61] Paek J Bai L Shin Y Kim H Kook JK Chang YH. Description of paenibacillus dokdonensis sp. Nov., a new bacterium isolated from soil. Int J systematic evolutionary Microbiol (2019) 71(3). doi: 10.1099/ijsem.0.004707 33595431

[62] Shin Y Paek J Kim H Kook JK Kim JS Kim SH. Absicoccus porci gen. Nov., sp. Nov., a member of the family erysipelotrichaceae isolated from pig faeces. Int J systematic evolutionary Microbiol (2020) 70(2):732–7. doi: 10.1099/ijsem.0.003803 31702538

[63] Seemann T. Prokka: rapid prokaryotic genome annotation. Bioinf (Oxford England) (2014) 30(14):2068–9. doi: 10.1093/bioinformatics/btu153 24642063

[64] Meier-Kolthoff JP Carbasse JS Peinado-Olarte RL Göker M. Tygs and lpsn: a database tandem for fast and reliable genome-based classification and nomenclature of prokaryotes. Nucleic Acids Res (2022) 50(D1):D801–d7. doi: 10.1093/nar/gkab902 PMC8728197 34634793

[65] Alanjary M Steinke K Ziemert N. Automlst: an automated web server for generating multi-locus species trees highlighting natural product potential. Nucleic Acids Res (2019) 47(W1):W276–w82. doi: 10.1093/nar/gkz282 PMC6602446 30997504

[66] Lee BH Chathuranga K Uddin MB Weeratunga P Kim MS Cho WK. Coptidis rhizoma extract inhibits replication of respiratory syncytial virus in vitro and in vivo by inducing antiviral state. J Microbiol (Seoul Korea) (2017) 55(6):488–98. doi: 10.1007/s12275-017-7088-x 28551874

[67] Kim JH Weeratunga P Kim MS Nikapitiya C Lee BH Uddin MB. Inhibitory effects of an aqueous extract from cortex phellodendri on the growth and replication of broad-spectrum of viruses in vitro and in vivo. BMC complementary Altern Med (2016) 16:265. doi: 10.1186/s12906-016-1206-x PMC4970287 27484768

[68] Ramakrishnan MA. Determination of 50% Endpoint titer using a simple formula. World J Virol (2016) 5(2):85. doi: 10.5501/wjv.v5.i2.85 27175354 PMC4861875

[69] Itoh Y Shinya K Kiso M Watanabe T Sakoda Y Hatta M. *In vitro* and in vivo characterization of new swine-origin H1n1 influenza viruses. Nature (2009) 460(7258):1021–5. doi: 10.1038/nature08260 PMC2748827 19672242

[70] Shim B-S Choi YK Yun C-H Lee E-G Jeon YS Park S-M. Sublingual immunization with M2-based vaccine induces broad protective immunity against influenza. PloS One (2011) 6(11):e27953. doi: 10.1371/journal.pone.0027953 22140491 PMC3227615

[71] Kim PI Jung MY Chang YH Kim S Kim SJ Park YH. Probiotic properties of lactobacillus and bifidobacterium strains isolated from porcine gastrointestinal tract. Appl Microbiol Biotechnol (2007) 74(5):1103–11. doi: 10.1007/s00253-006-0741-7 17136367

[72] Jung MY Cho JH Shin Y Paek J Park I-S Kim J-S. Peptoniphilus rhinitidis sp. Nov., isolated from specimens of chronic rhinosinusitis. Anaerobe (2014) 30:30–4. doi: 10.1016/j.anaerobe.2014.07.005 25094054

[73] Foligne B Nutten S Grangette C Dennin V Goudercourt D Poiret S. Correlation between in vitro and in vivo immunomodulatory properties of lactic acid bacteria. World J gastroenterol: WJG (2007) 13(2):236. doi: 10.3748/wjg.v13.i2.236 17226902 PMC4065951

