# Supplementary material for: The novel immunobiotic Clostridium butyricum S-45-5 displays broad-spectrum antiviral activity in vitro and in vivo by inducing immune modulation
Source: Front Immunol. 2023 Oct 10;14:1242183. doi: 10.3389/fimmu.2023.1242183 (PMC10595006; doi:10.3389/fimmu.2023.1242183)
Supplement: Supplementary file 1 [file DataSheet_1.pdf]

frontiers

Supplementary Material

The novel immunobiotic *Clostridium butyricum* S-45-5 displays broad-spectrum antiviral activity *in vitro* and *in vivo* by inducing immune modulation

Kiramage Chathuranga[1†], Yeseul Shin[2†], Md Bashir Uddin[1,3,4†], Jayoung Paek[2], W.A. Gayan Chathuranga[1], Yebin Seong[1], Lu Bai[2], Hongik Kim[5], Jeong Hwan Shin[6], Young-Hyo Chang[2]*, and Jong-Soo Lee[1]*

Correspondence: Young-Hyo Chang (yhchang@kribb.re.kr), and Jong-Soo Lee(jongsool@cnu.ac.kr)

```
5’GNCGGNGTGCTACCATGCAGTCGAGCGATGAAGCTCCTTCGGGAGTGGATTAGCGGCGGACGGGTGAGTAACACGTGGGTAACCTGCCTCATAGAGGGGAATAGCC
TTTCGAAAGGAAGATTAATACCGCATAAGATTGTAGTACCGCATGGTACAGCAATTAAAGGAGTAATCCGCTATGAGATGGACCCGCGTCGCATTAGCTAGTTGGTGAG
GTAACGGCTCACCAAGGCGACGATGCGTAGCCGACCTGAGAGGGTGATCGGCCACATTGGGACTGAGACACGGCCCAGACTCCTACGGGAGGCAGCAGTGGGGAATA
TTGCACAATGGGGGAAACCCTGATGCAGCAACGCCGCGTGAGTGATGACGGTCTTCGGATTGTAAAGCTCTGTCTTTAGGGACGATAATGACGGTACCTAAGGAGGAA
GCCACGGCTAACTACGTGCCAGCAGCCGCGGTAATACGTAGGTGGCAAGCGTTGTCCGGATTTACTGGGCGTAAAGGGAGCGTAGGTGGATATTTAAGTGGGATGTGA
AATACCCGGGCTTAACCTGGGTGCTGCATTCCAAACTGGATATCTAGAGTGCAGGAGAGGAAAGGAGAATTCCTAGTGTAGCGGTGAAATGCGTAGAGATTAGGAAGA
ATACCAGTGGCGAAGGCGCCTTTCTGGACTGTAACTGACACTGAGGCTCGAAAGCGTGGGGAGCAAACAGGATTAGATACCCTGGTAGTCCACGCCGTAAACGATGAA
TACTAGGTGTAGGGGTTGTCATGACCTCTGTGCCGCCGCTAACGCATTAAGTATTCCGCCTGGGGAGTACGGTCGCAAGATTAAAACTCAAAGGAATTGACGGGGGCCC
GCACAAGCAGCGGAGCATGTGGTTTAATTCGAAGCAACGCGAAGAACCTTACCTAGACTTGACATCTCCTGAATTACTCTGTAATGGAGGAAGCCACTTCGGTGGCAGG
AAGACAGGTGGTGCATGGTTGTCGTCAGCTCGTGTCGTGAGATGTTGGGTTAAGTCCCGCAACGAGCGCAACCCTTATTGTTAGTTGCTACCATTTAGTTGAGCACTCTA
GCGAGACTGCCCGGGTTAACCGGGAGGAAGGTGGGGATGACGTCAAATCATCATGCCCCTTATGTCTAGGGCTACACACGTGCTACAATGGTCGGTACAATGAGATGC
AACCTCGCGAGAGTGAGCAAACTATAAAACCGATCTCAGTTCGGATTGTAGGCTGAAACTCGCCTACATGAAGCTGGAGTTGCTAGTAATCGCGAATCAAATGTCGCGG
TGAATACGTTCCCGGGCCTTGTACACACGCCCGTCACACCATGAGAGTTGGCATACCAAGTTCGTGAGCTAACCGCAGAGCAGCGACCTATGGTAGGTCAGCGATTGNG
GTGAGNCGTAACAGGG-3’
```

Supplementary Figure S1.

RNA sequencing result of the *Clostridium butyricum* S-45-5. The isolated strain was identified by 16S rRNA gene analysis. To amplify 16S rDNA from purified isolates, polymerase chain reactions (PCRs) were carried out using forward primer 27F (5'-agagtttgatccctcag-3') and a reverse primer 1492R (5'-ggttaccttgttacgactt-3'). Amplified genes were identified through sequencing analysis by Biofact Co.Ltd.

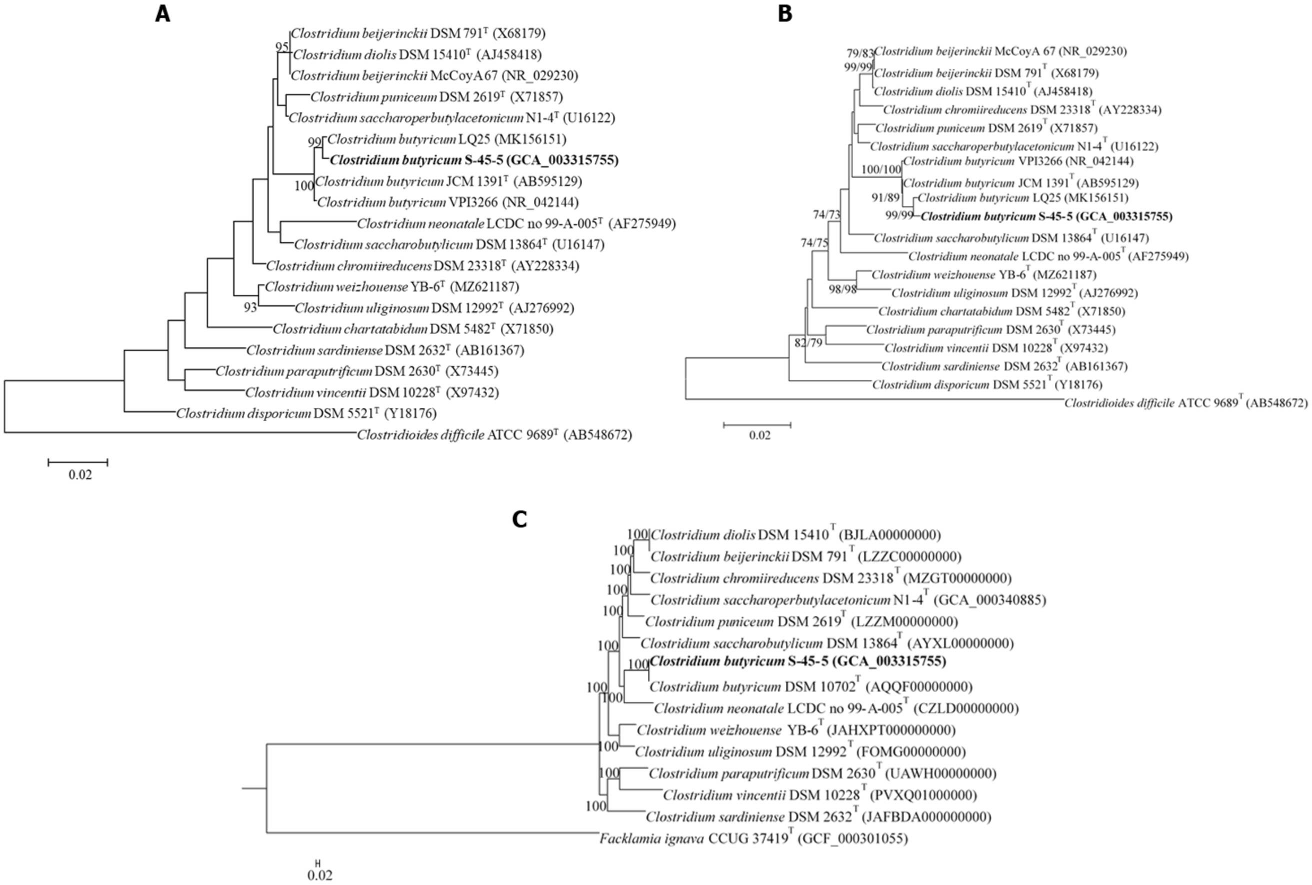

Supplementary Figure S2.

(A) Phylogenetic consensus tree based on 16S rRNA gene sequence of isolate *C. butyricum* S-45-5, reconstructed with the maximum-likelihood (ML), indicating the taxonomic positions of isolate and its close relatives. Bootstrap values (>70 %) calculated for 1000 subsets were shown at branch nodes. *Clostridioides difficile* ATCC 9689T served as an outgroup. Bar, 0.02 substitutions per nucleotide position. (B) Phylogenetic consensus tree based on 16S rRNA gene sequence of isolate *C. butyricum* S-45-5, reconstructed with the neighbor-joining (NJ) and the minimum-evolution (ME), indicating the taxonomic positions of isolate and its close relatives. Bootstrap values (>70%) calculated for 1000 subsets were shown at branch nodes. *Clostridioides difficile* ATCC 9689T served as an outgroup. Bar, 0.02 substitutions per nucleotide position. (C) Molecular phylogenetic analysis of isolate *C. butyricum* S-45-5 using default parameters (>100 core genes) by auto MLST. Bootstrap values (>70%) calculated for 1000 subsets were shown at branch nodes. *Facklamia ignava* CCUG 37419T was served as an outgroup. Bar, 0.02 substitutions per nucleotide position.

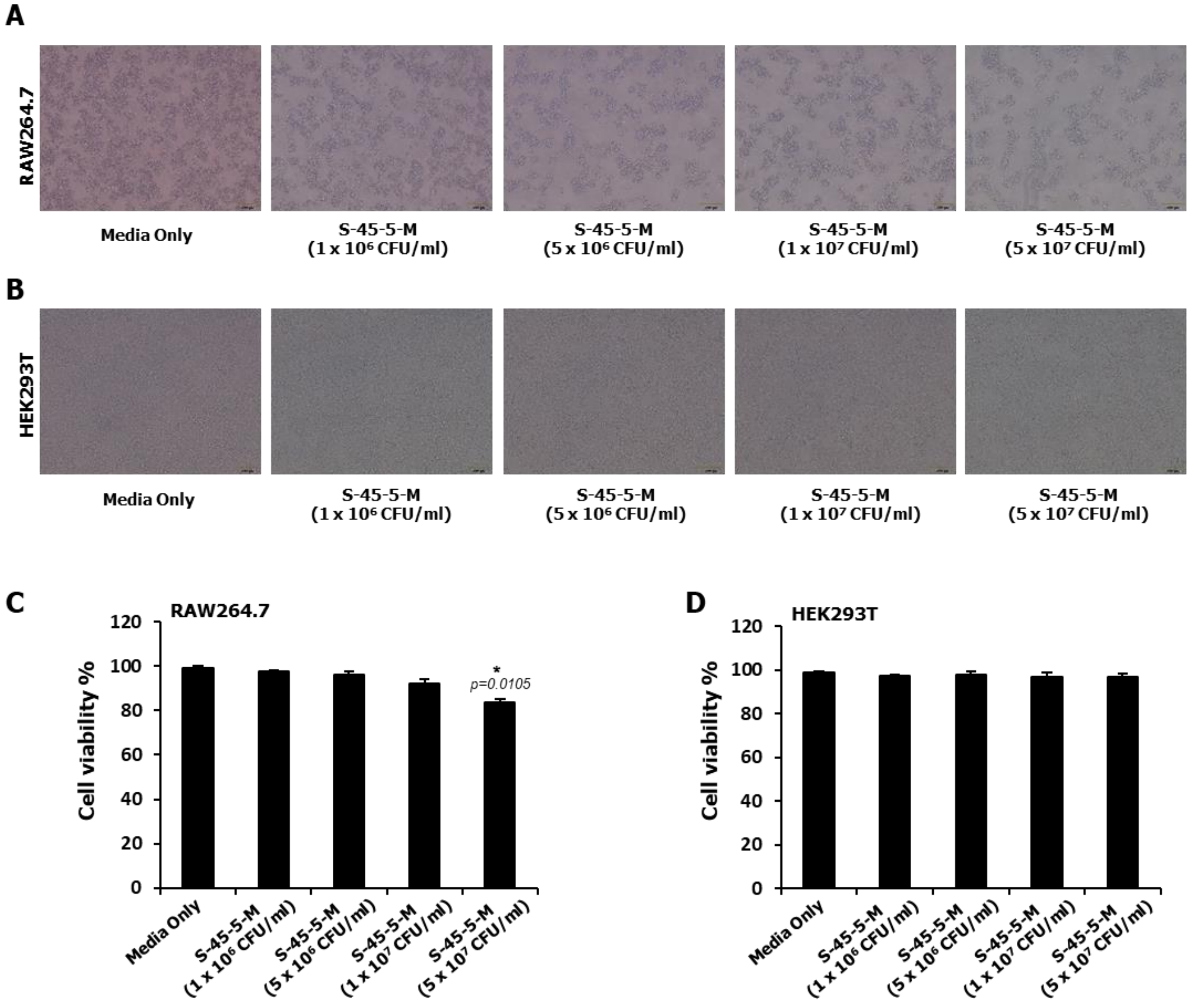

Supplementary Figure S3.

RAW264.7 cells or HEK293T cells were seeded in 12 well cell culture plates with the cell number of 3 x 10^5 cells/well. Twelve hours later, indicated colony-forming units (CFU) of *C. butyricum* S-45-5 were added to the cell medium. (A, B) At 24-hour post treatment images were obtained. (C, D) Cell viability was determined by trypan blue exclusion assay at the same time point. (S-45-5-M: *C. butyricum* S-45-5-Mix). Cell viability results are expressed as mean ± standard deviations (SD). Error bars indicate the range of values obtained from counting duplicates in three independent experiments (* $p < 0.05$ regarded as a significant difference).

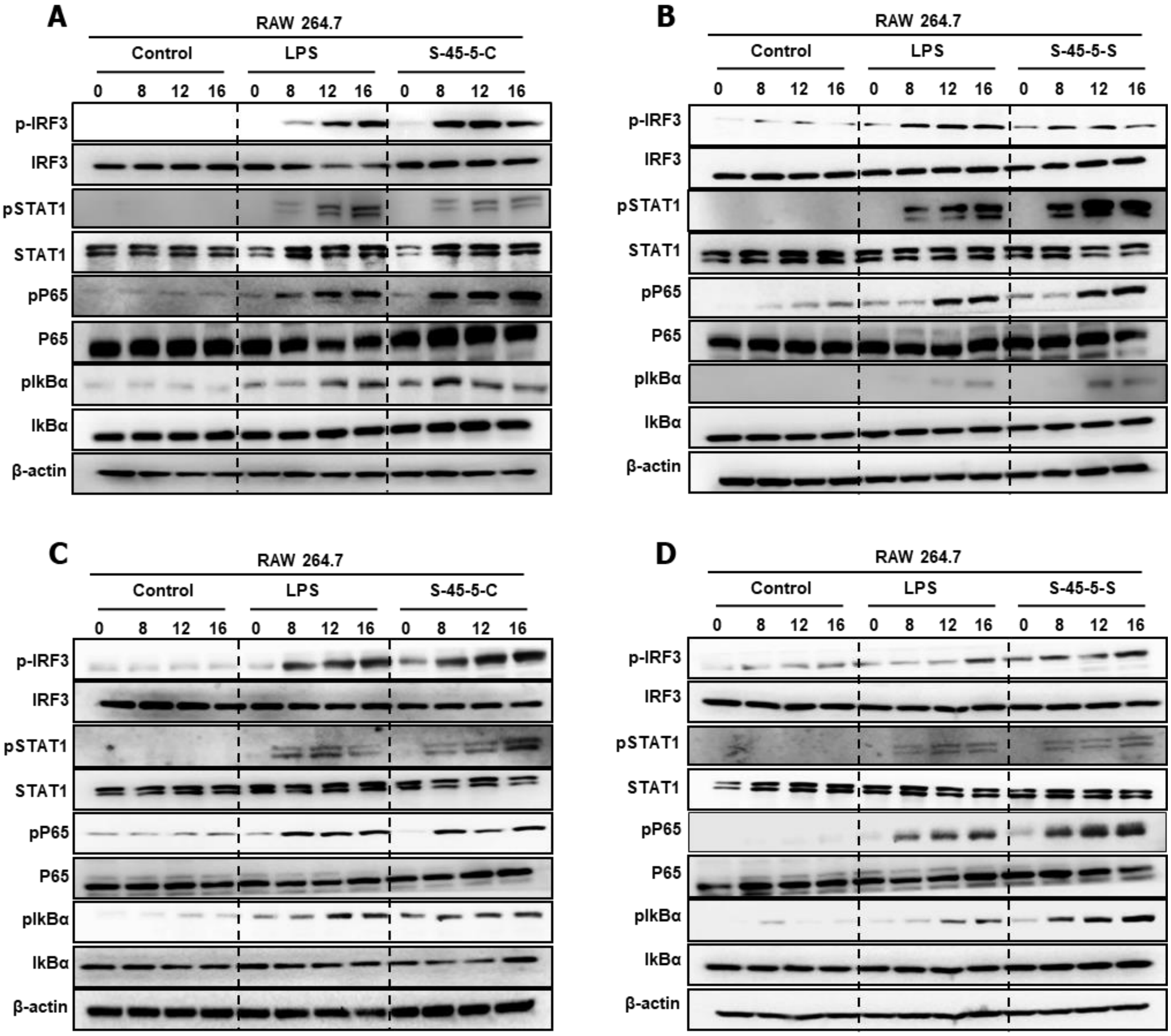

Supplementary Figure S4.

Activation of signal molecules present in Type-I IFN and NF-κB pathways were detected based on their phosphorylation status. RAW264.7 cells were treated with PBS, LPS (100 ng/ml) or *C. butyricum* S-45-5-Cell (1×10^6 CFU/ml) or *C. butyricum* S-45-5-Sup (1×10^6 CFU/ml) and cells were harvested at 0, 8, 12 and 16 hpt. Expression of the phosphorylated and non-phosphorylated forms of IRF3, STAT1, p65, and IkBα was analyzed time-dependently by immunoblotting. β-actin was used to confirm equal loading of proteins. A (*C. butyricum* S-45-5-Cell), B (*C. butyricum* S-45-5-Sup) (Technical repeat 2 results), C (*C. butyricum* S-45-5-Cell), D (*C. butyricum* S-45-5-Sup) (Technical repeat 3 results).

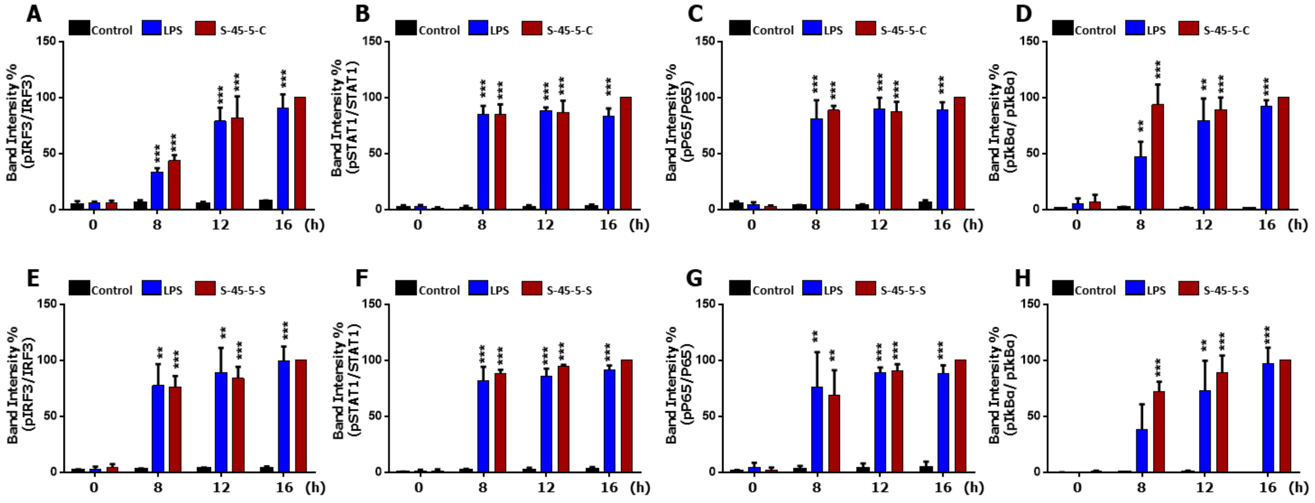

Supplementary Figure S5.

Immunoblot band intensities of pIRF3, pSTAT1, pP65, and pIκBα were calculated using imageQuant LAS 4000 mini software. The phosphorylated protein band was normalized with its respective normal protein band intensity. Band intensity percentage (16 h time point of S-45-5 set as 100%) results are expressed as mean ± standard deviations (SD). Error bars indicate the range of values obtained from three independent experiments (Figure 2B-C, Supplementary Figure 4A-D) (A-D, *C. butyricum* S-45-5-Cell, E-H, *C. butyricum* S-45-5-Sup) (** $p < 0.01$, *** $p < 0.001$ regarded as a significant difference). (S-45-5-C: *C. butyricum* S-45-5-Cell, S-45-5-S *C. butyricum* S-45-5-Sup).

A

Day	PBS		H1N1		S-45-5 Cell			S-45-5 Sup			S-45-5 Mix			S-45-5 IFN-β		
	Mean	SD	Mean	SD	Mean	SD	Significant	Mean	SD	Significant	Mean	SD	Significant	Mean	SD	Significant
1	100	0	100	0	100	0		100	0		100	0		100	0	
2	104.0039	1.503692	98.62049	1.233611	98.2188	1.051993		91.64894	3.202566	*	95.97871	2.693312		94.83488	2.960346	
3	99.64742	3.707882	93.94147	1.422014	94.31966	2.284974		93.76262	5.618426		93.17609	4.60984		95.63188	5.378263	
4	103.424	1.778368	91.5405	1.570954	94.64347	1.260846	*	93.86357	4.432463		95.48009	2.610809		93.94106	4.722282	
5	105.0088	1.714898	88.53168	2.460854	93.39538	2.342229	*	91.42929	3.737944		92.79456	4.414179		92.88586	3.748953	
6	99.28499	1.855969	85.67216	3.510731	92.10305	2.463625	*	92.11883	6.862363		91.32144	7.075628		93.16488	4.711483	
7	103.4207	1.647183	83.52134	2.744004	87.10225	3.05569		90.14635	7.390393		90.71423	7.210253		92.74355	8.48865	
8	102.0879	3.03704	79.73915	4.301113	88.91248	6.31117		88.66495	8.783946		88.64856	9.43495		91.26819	8.142756	
9	101.2198	3.940978	78.43733	4.025306	88.82505	7.650001		87.34402	9.599289		87.04358	7.292349		93.15732	3.834199	*
10	99.93129	3.390288	78.99764	5.214032	89.21484	8.592044		92.05403	10.02759		94.19553	2.674113	*	94.41358	5.763445	*
11	101.8067	5.115463	76.8452	3.034532	87.13392	8.436063		91.95508	10.06093		94.13741	2.785791	*	94.98975	4.460466	*
12	102.7175	4.571857	77.98568	3.353211	94.64856	4.977226	*	93.19496	10.82187		96.1027	3.015811	*	94.57008	3.131052	*
13	103.1554	3.30456	78.85322	4.663061	97.62181	2.833417	*	97.90397	1.435808	*	98.16636	3.564618	*	97.23169	3.374127	*

B

Day	PBS		H3N2		S-45-5 Cell			S-45-5 Sup			S-45-5 Mix			S-45-5 IFN-β		
	Mean	SD	Mean	SD	Mean	SD	Significant	Mean	SD	Significant	Mean	SD	Significant	Mean	SD	Significant
1	100	0	100	0	100	0		100	0		100	0		100	0	
2	97.93919	0.7289436	96.30545	0.6230653	97.58063	2.4864		97.19714	1.518929		95.92619	1.430485		99.49355	2.769705	
3	98.86887	1.921113	94.67005	1.948315	97.66923	3.646497		96.62416	1.112845		94.6077	3.377521		100.6302	3.83274	
4	100.7146	2.067173	93.79419	3.502308	97.06611	4.726394		94.33588	1.454781		93.30072	4.947278		98.60088	4.032623	
5	101.1907	1.921208	90.14584	5.516561	95.01874	6.514047		92.24561	3.456598		90.46125	5.134038		98.08046	5.036587	
6	102.9892	1.519387	87.37349	7.650084	93.34343	7.697055		91.02279	6.557214		90.17062	7.578773		96.72352	6.16074	
7	101.6849	1.285927	82.50835	7.392198	91.24674	9.117389		89.33415	5.713017		88.7823	7.725627		92.49712	5.509496	
8	102.3287	1.494883	81.90999	5.221354	90.24814	9.231503		87.79335	6.551573		87.94066	9.523476		94.01028	7.568208	
9	104.0967	1.081403	77.91548	6.001976	90.43963	11.27194		86.93291	8.635462		92.16087	7.881972		94.54747	10.85646	
10	103.9926	1.767157	77.59879	4.814394	94.69328	11.46591		85.83219	8.721869		89.55014	8.29568		92.60189	11.03828	
11	103.6152	1.000771	76.59187	3.530916	101.768	2.673264	*	83.96742	11.20969		90.27489	8.81633		97.35837	5.355584	*
12	103.4086	1.252783	76.50529	4.369424	101.8061	3.048892	*	93.70305	3.016355		97.22658	1.807316	*	101.0723	3.860198	*
13	105.3943	1.063128	76.56615	5.715469	102.9573	3.937316	*	94.82299	2.500694		99.90253	4.10773	*	101.2514	4.520144	*

C

Day	PBS		H9N2		S-45-5 Cell			S-45-5 Sup			S-45-5 Mix			S-45-5 IFN-β		
	Mean	SD	Mean	SD	Mean	SD	Significant	Mean	SD	Significant	Mean	SD	Significant	Mean	SD	Significant
1	100	0	100	0	100	0		100	0		100	0		100	0	
2	99.68385	1.294653	97.31909	0.6764152	97.98547	1.737029		98.23751	0.8228639		96.55152	2.063488		98.81599	1.512507	
3	100.0434	0.9674618	94.26427	2.306568	95.53967	2.362307		95.80997	1.734265		94.826	3.889638		99.80183	2.540013	*
4	100.14	0.8026579	89.13982	1.170721	92.49158	1.729405		92.43399	1.270777	*	93.45083	5.342535		98.5025	2.658692	*
5	100.6028	1.225845	84.74516	1.715179	89.17064	1.147092	*	90.40976	2.830007	*	90.51053	5.409178		97.93371	4.415377	*
6	100.5423	1.331248	80.72176	4.01806	87.0692	1.6598	*	87.75789	4.794542		90.3916	8.071802		96.36852	5.442514	*
7	100.8754	1.318635	80.02673	3.1525	86.62446	4.563458	*	88.04528	5.137773		89.08768	8.321765		92.81817	6.298597	*
8	101.0432	1.329386	77.77589	2.065275	86.92544	7.566965		88.46043	6.663013		88.2101	10.00366		93.31111	11.67007	
9	101.0604	1.505786	75.79811	1.833194	90.67948	3.493809		89.43857	7.990681		92.28023	8.074899		96.06741	7.515854	*
10	102.7307	1.666425	74.62377	0	91.3674	3.468644	*	90.13708	8.983928		90.29366	8.922382		96.33795	4.877106	
11	103.1445	1.673243			94.08945	1.78976		96.30263	5.020638		90.92047	9.506289		97.0387	4.367816	
12	102.9579	1.612838			94.18803	2.52469		98.17591	4.427733		97.43616	1.261535		99.99184	3.598008	
13	103.4903	2.059858			95.55286	2.601823		100.2868	2.631272		100.0806	4.023767		100.232	2.677874	

Supplementary Figure S6.
Six-week-old female BALB/c mice were orally administrated with *C. butyricum* S-45-5-Mix (5×10^7 CFU) (n=6), or *C. butyricum* S-45-5-Cell (5×10^7 CFU) (n=6) or *C. butyricum* S-45-5-Sup (5×10^7 CFU) (n=6) in a total volume of 100 µl and control mice (PBS, Virus only, IFN-β) with 100 µl of PBS daily for 21 days as separate groups. Twelve hours before infection, mice in the positive control group were intranasally inoculated with 1000 units of rmIFN-β. Except for the PBS group, all mice were intranasally infected with $2MLD_{50}$ of H1N1, H3N2, and H9N2 influenza A subtypes. Except for the PBS group, all mice were intranasally infected with $2MLD_{50}$ of H1N1 or, H3N2, or H9N2 influenza A subtypes. Average body weight percentages and standard deviation are shown in the table. * $p < 0.05$ regarded as a significant difference (two-tailed student's t-test).

Supplementary Table S1. Genomic characteristics of *C. butyricum* S-45-5

Attribute	S-45-5
Accession no.	GCA_003315755
Coverages (×)	263
N50 length (bp)	3,810,128
Genome size (bp)	4,588,510
DNA G+C content (mol%)	28.7
No. of contigs	2
No. of CDS	4,077
rRNAs	28
tRNAs	87

Supplementary Table S2. Average Nucleotide Identity (ANI), and digital DNA-DNA hybridization (dDDH) values for *C. butyricum* S-45-5 and related strains in the species *C. butyricum*

No.	GenBank Accession	Strain	dDDH (%)	ANI (%)	Size (Mp)	WGS Accession	Assembly Level	Release Date
**1**	**GCA_00331**	**S-45-5**	**100**	**100**	**4.6**	**-**	**Complete**	**Jul, 2018**
**2**	GCA_00514	4-1	96.3	99.6	4.6	-	Complete	May, 2019
**3**	GCA_01311	16-3	96.3	99.6	4.6	-	Complete	May, 2020
**4**	GCA_01314	DJ064	96.3	99.6	4.6	JABFUG01	Contig	May, 2020
**5**	GCA_01413	DJ046	96.3	99.6	4.7	JACJHS01	Contig	Aug, 2020
**6**	GCA_01413	DJ075	96.3	99.6	4.6	JACGZE01	Contig	Aug, 2020
**7**	GCA_02665	QXYZ514	96.3	99.6	4.6	-	Complete	Dec, 2022
**8**	GCA_02762	LV1	96.3	99.6	4.6	-	Complete	Jan, 2023
**9**	GCA_03038	DKU-11	96.3	99.6	4.6	-	Complete	Jul, 2023
**10**	GCA_00087	HM-68	96.2	99.6	4.6	JXBT01	Contig	Feb, 2015
**11**	GCA_00799	NBRC 3315	96.2	99.5	4.5	BKBB01	Contig	Jul, 2019
**12**	GCA_01423	DJ013	96.2	99.6	4.5	JAAZWP0	Contig	Aug, 2020
**13**	GCA_02596	NPLps03.gc	96.2	99.6	4.5	JAOZWJ01	Contig	Nov, 2022
**14**	GCA_00345	AF25-25	96.1	99.6	4.5	QRUG01	Scaffold	Sep, 2018
**15**	GCA_01735	DS501	95.7	99.5	3.9	-	Chromosome	Mar, 2021
**16**	GCA_03029	SJ1	95.7	99.5	3.9	-	Complete	Jun, 2023
**17**	GCA_02767	AM32-13	95.3	99.6	4.5	JAQEBU01	Scaffold	Jan, 2023
**18**	GCA_02767	AM21-3LB	95.3	99.3	4.5	JAQDZY01	Scaffold	Jan, 2023
**19**	GCA_01566	1001311H_170123_	95.0	99.4	4.5	JADPEK01	Scaffold	Nov, 2020
**20**	GCA_01566	D52t1_170925_F3	94.4	99.4	4.6	JADPLV01	Scaffold	Nov, 2020
**21**	GCA_02821	D52st1_A4_D52t1_	94.3	99.4	4.6	JAQLDD01	Scaffold	Jan, 2023
**22**	GCA_01756	GGCC_0151	94.2	99.3	3.8	JABFCF01	Contig	Mar, 2021
**23**	GCA_03003	MALS002	94.1	99.4	4.6	JALGRX01	Contig	May, 2023
**24**	GCA_02821	BSD3178071175st2	93.9	99.3	4.7	JAQLDE01	Scaffold	Jan, 2023
**25**	GCA_00965	CFSA3987	93.8	99.3	4.7	-	Complete	Nov, 2019
**26**	GCA_00965	CFSA3989	93.8	99.3	4.7	-	Complete	Nov, 2019
**27**	GCA_93275	DSM 107392	93.8	99.3	4.6	CAKODI01	Contig	Mar, 2022
**28**	GCA_90236	MGYG-HGUT-00014	93.6	99.3	4.6	CABIVR01	Scaffold	Aug, 2019
**29**	GCA_01567	D33t1_170424_B3	93.5	99.2	4.6	JADPCQ01	Scaffold	Nov, 2020

**30**	GCA_90520	SRR7721873-bin.10	93.4	99.2	4.6	CAJKPI01	Contig	Feb, 2021
**31**	GCA_01566	1001287H_170206_	93.0	99.2	4.5	JADPAI01	Scaffold	Nov, 2020
**32**	GCA_02768	AF13-51	93.0	99.2	4.7	JAQCUK01	Scaffold	Jan, 2023
**33**	GCA_02768	AF13-166.0	93.0	99.2	4.6	JAQCUF01	Scaffold	Jan, 2023
**34**	GCA_90216	4928STDY7387863	93.0	99.2	4.6	CABHIF01	Scaffold	Jul, 2019
**35**	GCA_00141	SU1	92.9	99.2	4.9	LIDW01	Contig	Oct, 2015
**36**	GCA_02439	LCL-063	92.9	99.2	4.5	JAJDMU0	Scaffold	Jul, 2022
**37**	GCA_00037	60E.3	92.8	99.2	4.6	AGYK01	Scaffold	Apr, 2013
**38**	GCA_00145	NEC8	92.7	99.2	4.8	CBYK01	Contig	Feb, 2014
**39**	GCA_02768	AF13-10-6.0-r	92.6	99.2	4.6	JAQCUC0	Scaffold	Jan, 2023
**40**	GCA_00094	CWBI1009	92.4	99.1	4.5	ASPQ01	Contig	Mar, 2015
**41**	GCA_00987	BIOML-A2	92.4	99.2	4.7	WWTZ01	Contig	Jan, 2020
**42**	GCA_02439	LCL-155	92.1	99.2	4.6	JAJDMT01	Scaffold	Jul, 2022
**43**	GCA_01814	CBUT	91.9	99.1	4.5	-	Complete	Apr, 2021
**44**	GCA_93797	SRR3546782_bin.56	91.9	99.1	4.5	CALJIR01	Contig	Jan, 2023
**45**	GCA_00040	DSM 10702	91.8	99.1	4.6	AQQF01	Contig	Jun, 2013
**46**	GCA_00674	NBRC 13949	91.8	99.0	4.7	-	Complete	Jun, 2019
**47**	GCA_00799	NBRC 13949	91.8	99.1	4.6	BKBD01	Contig	Jul, 2019
**48**	GCA_01413	DSM 10702	91.8	99.1	4.7	-	Complete	Aug, 2020
**49**	GCA_02439	KZ-1890	91.8	99.1	4.6	JAJDMV01	Scaffold	Jul, 2022
**50**	GCA_02439	CFSA-TJ-E	91.8	99.1	4.7	-	Complete	Jul, 2022
**51**	GCA_01101	H102020561	91.7	99.1	4.7	SGLE01	Contig	Feb, 2020
**52**	GCA_01101	H102020560	91.7	99.1	4.7	SGLF01	Contig	Feb, 2020
**53**	GCA_02007	LY33	91.7	99.0	4.6	JAIQXW01	Scaffold	Sep, 2021
**54**	GCA_02439	KZ-1886	91.7	99.1	4.6	JAJDMW0	Scaffold	Jul, 2022
**55**	GCA_00035	DKU-01	91.4	99.0	4.5	APKZ01	Contig	Apr, 2013
**56**	GCA_00145	KNU-L09	91.4	99.0	4.6	-	Complete	Dec, 2015
**57**	GCA_00146	JKY6D1	91.4	99.0	4.6	-	Complete	Dec, 2015
**58**	GCA_00164	TOA	91.4	99.0	4.6	-	Complete	May, 2016
**59**	GCA_00514	29-1	91.3	99.0	4.6	JALAYW01	Contig	Mar, 2022
**60**	GCA_01337	HYCB	91.3	99.0	4.5	JABXWN0	Contig	Jun, 2020
**61**	GCA_03037	ET61	91.2	99.0	4.4	JAUDBX01	Contig	Jun, 2023
**62**	GCA_03037	Avi11	91.1	99.0	4.5	JAUDDA0	Contig	Jun, 2023
**63**	GCA_00076	INCQS635	90.3	99.0	4.4	JRMA01	Contig	Oct, 2014
**64**	GCA_00017	5521	85.7	98.4	4.5	ABDT01	Contig	Apr, 2008
**65**	GCA_00018	BoNT E BL5262	85.7	98.4	4.8	ACOM01	Scaffold	May, 2009
**66**	GCA_01101	ATCC 43755	85.6	98.4	4.5	SWXL01	Contig	Feb, 2020
**67**	GCA_01101	BL-5262-9RE	85.5	98.4	4.7	SWZQ01	Contig	Feb, 2020
**68**	GCA_00188	CDC_51208	85.3	98.4	4.6	-	Complete	Nov, 2016
**69**	GCA_00384	JKT37	84.2	98.3	4.2	RQKF01	Scaffold	Nov, 2018
**70**	GCA_00973	S3	83.4	98.2	4.4	WOFV02	Scaffold	Jan, 2020
**71**	GCA_01991	GD1_1	82.9	98.0	4.5	JAIOKK01	Scaffold	Sep, 2021
**72**	GCA_00050	DORA_1	82.6	98.1	4.0	AZLX01	Scaffold	Dec, 2013
**73**	GCA_01314	DJ065	82.6	98.1	4.7	JABFUH01	Contig	May, 2020
**74**	GCA_00799	NBRC 3858	82.4	98.1	4.5	BKBC01	Contig	Jul, 2019
**75**	GCA_01555	1001713B170214_1	81.3	97.8	4.6	JADNPC01	Scaffold	Nov, 2020
**76**	GCA_02821	1001713st1_G1_10	81.3	97.8	4.6	JAQLDF01	Scaffold	Jan, 2023
**77**	GCA_94738	B111	80.9	97.8	4.4	CANCWB0	Contig	Apr, 2023
**78**	GCA_94738	B067	80.2	97.8	3.7	CANCWA0	Contig	Apr, 2023
**79**	GCA_00294	300064	79.9	97.7	4.7	LRDH01	Contig	Feb, 2018
**80**	GCA_01837	L3_063_040G1_das	79.8	97.8	4.5	JAHAII01	Contig	May, 2021
**81**	GCA_00042	AGR2140	79.3	97.7	4.6	AUJN01	Scaffold	Jul, 2013
**82**	GCA_00078	NOR 33234	78.8	97.6	4.9	JSEG01	Contig	Nov, 2014

Supplementary Table S3. Mouse primer sets used to confirm mRNA expression

Gene	Primers	
	Forward	Reverse
**IFN-β**	5'-TCCAAGAAAGGACGAACATTCG-3'	5'-TGCGGACATCTCCCACGTCAA-3'
**IFN-α**	5'-CTCTCCTGCCTGAAGGACAGGAAG-3'	5'-GGTGGAGGTCATTGCAGAATGAGT-3'
**IL-6**	5'-TCCATCCAGTTGCCTTCTTGG-3'	5'-CCACGATTTCCCAGAGAACATG-3'
**ISG-15**	5'-CAATGGCCTGGGACCTAAA-3'	5'-CTTCTTCAGTTCTGACACCGTCAT-3'
**ISG-20**	5'-AGAGATCACGGACTACAGAA-3'	5'-TCTGTGGACGTGTCATAGAT-3'
**ISG-56**	5'-AGAGAACAGCTACCACCTTT-3'	5'-TGGACCTGCTCTGAGATTCT-3'
**OAS-1β**	5'-GAGGCGGTTGGCTGAAGAGG-3'	5'-GAGGAAGGCTGGCTGTGATTGG-3'
**GBP-1**	5'-AAAAACTTCGGGGACAGCTT-3'	5'-CTGAGTCACCTCATAAGCCAAA-3'
**Mx1**	5'-ACAAGCACAGGAAACCGTATCAG-3'	5'-AGGCAGTTTGGACCATCTTAGTG-3'
**GAPDH**	5'-TGACCACAGTCCATGCCATC-3'	5'-GACGGACACATTGGGGGTAG-3'

Supplementary Table S4. *In vivo* (mouse) experiment summary

Group	Treatment	Administration Dose	Administration Route & Duration	Challenged Virus	Experiment
Negative Control (n=6)	PBS	-	Oral for 21 days	H1N1	Survival rate
Positive Control (n=6)	IFNβ	1000U	IN/12h prior infection		
S-45-5 (n=18) (Cell, Sup, Mix)	S-45-5	$5x10^7$(cfu/mL)	Oral for 21 days		
Negative Control (n=6)	PBS	-	Oral for 21 days	H3N2	Survival rate
Positive Control (n=6)	IFNβ	1000U	IN/12h prior infection		
S-45-5 (n=18) (Cell, Sup, Mix)	S-45-5	$5x10^7$(cfu/mL)	Oral for 21 days		
Negative Control (n=6)	PBS	-	Oral for 21 days	H9N2	Survival rate
Positive Control (n=6)	IFNβ	1000U	IN/12h prior infection		
S-45-5 (n=18) (Cell, Sup, Mix)	S-45-5	$5x10^7$(cfu/mL)	Oral for 21 days		
Negative Control (n=6) 3dpi (n=3), 5dpi (n=3)	PBS	-	Oral for 21 days	H1N1	Lung titration Histopathology
Positive Control (n=6) 3dpi (n=3), 5dpi (n=3)	IFNβ	1000U	IN/12h prior infection		Lung titration Histopathology
S-45-5 (n=18) (Cell, Sup, Mix)	S-45-5	$5x10^7$(cfu/mL)	Oral for 21 days		Lung titration Histopathology
